# Supplementary material for: Identification of host–pathogen-disease relationships using a scalable multiplex serology platform in UK Biobank
Source: Nat Commun. 2022 Apr 5;13:1818. doi: 10.1038/s41467-022-29307-3 (PMC8983701; doi:10.1038/s41467-022-29307-3)
Supplement: Supplementary file 1 — Supplementary Information [file 41467_2022_29307_MOESM1_ESM.pdf]

Identification of host-pathogen-disease relationships using a scalable Multiplex Serology platform in UK Biobank

Alexander J Mentzer†, Nicole Brenner†, Naomi Allen, Thomas J Littlejohns, Amanda Y Chong, Adrian Cortes, Rachael Almond, Michael Hill, Simon Sheard, Gil McVean, UKB Infection Advisory Board, Rory Collins, Adrian VS Hill, Tim Waterboer

Supplementary Methods and Results

This section describes the development, validation and application of the Multiplex Serology assay applied to 9,695 participants from UK Biobank (UKB), as well as demographic and genetic association analyses.

Contents

1. UK Biobank Infectious Disease Working Group 3

2. Infectious agents, antigen selection and prioritisation 3

3. The Multiplex Serology protocol 3

4. Assay validation 6

5. UK Biobank Multiplex Serology quality control 7

6. Multiplex Serology seroprevalence calculations from antigen reactivity data 7

7. UK Biobank Data 9

8. Testing for association between demographic variables and infectious agent serostatus 10

9. Genetic association tests in UKB 10

10. Supplementary Figures 12

Supplementary Figure 1: Proposed steps of validation for infectious agents of highlighted relevance to public health. 12

Supplementary Figure 2: Quality control of automatically GC-coupled magnetic bead sets. 13

Supplementary Figure 3: Comparison of statistical characteristics (specificity, sensitivity and Cohen's kappa) in Multiplex Serology on non-magnetic (blue) vs. magnetic (orange) beads for 10 infectious agents. 15

Supplementary Figure 4: Assessment of batch effects in the measurements of antibody responses to all 45 antigens in the UKB panel. 20

Supplementary Figure 5: Seroreactivity (MFI) of one control serum across all antigens and plates per assay week. 21

Supplementary Figure 6: Correlations between log-transformed antibody responses against beta herpesviridae antigens measured on the UKB Multiplex Serology platform. 22

Supplementary Figure 7: Associations between imputed autosomal variants and $\log_{10}$ transformed quantitative responses against EBV VCAp18 antigen in 9,611 UKB participants. 23

11. Supplementary Tables 24

Supplementary Table 1: Infectious agents considered and eventually taken forward for development on the Multiplex Serology platform. 24

Supplementary Table 2: Characteristics of infectious agent specific antigens used on the Multiplex Serology platform. 26

Supplementary Table 3: Intra-class correlation coefficients (ICC) comparing Multiplex Serology assays on non-magnetic and magnetic beads. 28

Supplementary Table 4: Correlation between tested bridging panel samples (n=182) on multiple assay days for each antigen. 29

Supplementary Table 5: Coefficients of variation (CV) for blind spiked duplicates (n=107) from samples from UKB. 30

Supplementary Table 6: Seroconversion and reversion rates estimated from 277 individuals with samples available at both baseline and repeat assessment encounters. 31

Supplementary Table 7: Unadjusted seroprevalence estimates and 95% confidence intervals for all antigens tested using the Multiplex Serology platform in the 9,695 individuals from UKB overall, and stratified by sex. 32

Supplementary Table 8: Crude seroprevalence estimates and adjusted odds ratio of antibody seroprevalence in the UKB subset. 33

Supplementary Table 9: Unadjusted seroprevalence estimates and adjusted odds ratios of infectious agent seroprevalence in the UKB sample clustered by age intervals and stratified by sex. 34

Supplementary Table 10: Unadjusted seroprevalence estimates and adjusted odds ratio of seroprevalence for multiple infectious agents in the UKB subset clustered by self-reported ethnicity and stratified by sex. 35

Supplementary Table 11: Unadjusted seroprevalence estimates and adjusted odds ratios of antibody prevalence against all tested infections in the UKB sample clustered by LSP and stratified by sex. 36

Supplementary Table 12: Unadjusted seroprevalence estimates and adjusted odds ratios of antibody prevalence in the UKB sample clustered by sex and Townsend deprivation index quartile. 38

Supplementary Table 13: Characteristics of human genetic variants available for analysis in the UKB subset. 39

Supplementary Table 14: HLA alleles associated with tested quantitative antibody responses. 39

12. References 40

1. *UK Biobank Infectious Disease Working Group*

Individuals were invited to participate in the Working Group to discuss selection of infectious agents of importance and advice on appropriate assay methodology. Participating individuals included:

**Individual**	**Affiliations**
Prof Adrian Hill (chair)	Jenner Institute, University of Oxford, UK
Prof Allison Aiello	University of North Carolina at Chapel Hill, USA
Prof Charles Bangham	Department of Medicine, Imperial College London, UK
Prof Ray Borrow	Public Health England, Manchester, UK
Prof Judy Breuer	Division of Infection and Immunity, University College London, UK
Dr Tim Brooks	Special Pathogens Reference Unit, Public Health England, UK
Prof Silvia Franceschi	Centro di Riferimento Oncologico, Italy
Dr Effrossyni Gkrania-Klotsas	University of Cambridge, Cambridge, UK
Prof Brian Greenwood	London School of Hygiene and Tropical Medicine, London, UK
Prof Paul Griffiths	Royal Free Hospital, London, UK
Dr Edward Guy	Public Health Wales, UK
Dr Katie Jeffery	John Radcliffe Hospital, Oxford, UK
Dr Dominic Kelly	Oxford Vaccine Group, University of Oxford, UK
Prof Paul Klenerman	Medawar Building, University of Oxford, UK
Dr Fiona van der Klis	National Institute for Public Health and Environment, Netherlands
Prof Julian Knight	Wellcome Centre for Human Genetics, University of Oxford, UK
Prof Andrew McMichael	University of Oxford, UK
Dr Alex Mentzer (writing group)	Wellcome Centre for Human Genetics, University of Oxford, UK
Dr Vivek Naranbhai	Wellcome Centre for Human Genetics, University of Oxford, UK
Dr Richard Pebody	Immunisation and Countermeasures Division, National Infections Service, Public Health England, UK
Prof Tim Peto	Experimental Medicine Division, University of Oxford, UK
Prof Andrew J Pollard	Oxford Vaccine Group, Department of Paediatrics, University of Oxford, UK
Prof Thomas Schulz	University of Hannover, Germany
Dr Kate Soldan	Public Health England, UK
Dr Graham Taylor	Imperial College London, UK
Prof Greg Towers	University College London, UK
Dr Massimo Tommasino	International Agency for Research on Cancer, Lyon, France
Prof Robin Weiss	Division of Infection and Immunity, University College London, UK
Dr Denise Whitby	AIDS and Cancer Virus Program, Frederick National Laboratory for Cancer Research, USA
Prof Chris Wild	International Agency for Research on Cancer, Lyon, France
Dr David Wyllie	Jenner Institute, University of Oxford, UK

2. *Infectious agents, antigen selection and prioritisation*

The Working Group were asked to suggest a range of infectious agents that were linked with the development of chronic disease and of likely relevance for UK public health. This included a) every viral or bacterial pathogen confirmed to be associated with cancer development (except several rare high-risk Human Papillomavirus types), b) all Human Herpesviruses which are known to establish life-long infections, c) several Human Polyomaviruses known or suspected to be involved in cancer development, and d) additional pathogens based on specific recommendations by the Working Group. A full list of infectious agents considered for inclusion in the Multiplex Serology panel are described in Supplementary Table 1 listing reasons for their suggestion and for their final in- or ex-clusion from the panel. Vaccine preventable diseases were ultimately excluded because it was not thought that there would be sufficient information available on numbers of doses given that may confound the antibody response levels detected.

3. *The Multiplex Serology protocol*

We used a standardised approach to validate the Multiplex Serology panel against established reference assays for each infectious agent where possible (Supplementary Figure 1). The development and protocol of Multiplex Serology has been described by Waterboer *et al*[1, 2] for human papillomavirus (HPV) serology, and is based on previously developed methods for bacterial recombinant expression of glutathione S-transferase (GST)-fusion

proteins as antigens[3, 4]. Since 2005, Multiplex Serology has been used in many seroepidemiological studies not only to measure antibodies against HPV but also against a magnitude of other infectious agents. In this work, we transferred the methodology from non-magnetic to magnetic beads to allow for high-throughput testing of large studies such as the UK Biobank (n~500,000 participants). In the following, we describe the changes to the previously used and published protocol for Multiplex Serology on magnetic beads.

Coupling of glutathione-casein (GC) to magnetic Luminex beads

Glutathione-casein (GC) was produced as described[1], and coupled to magnetic beads (MagPlex microspheres, Luminex) analogous to the previously described manual protocol for coupling of non-magnetic beads (SeroMAP, Luminex). In this work, we transferred the manual coupling protocol for non-magnetic beads to a KingFisher Flex device (Thermofisher 5400640) to allow for automatisation of the GC coupling to magnetic beads. The KingFisher Flex device uses a magnetic tip head to transfer the beads between different deep-well plates containing the coupling reagents. The device supports incubation and mixing phases fully replacing the usage of a centrifuge and shaker during the manual coupling procedure. For each of the 46 bead sets, 4 ml of beads (n= $50*10^6$ / bead set) were coupled with GC using the 24-tip-magnetic head, and 24-deep-well plates (KF Flex 24-deep well plate, 94040470 ThermoFisher). The 46 bead sets were coupled with GC in two batches (I n=24, II n=22) on two subsequent days three months before the study samples were analysed with Multiplex Serology.

<u>Protocol:</u>

Automatic coupling was performed with a KingFisher Flex. This device is able to mix, collect, and transfer magnetic beads between 96- or 24-well plates using a magnetic tip head. For coupling of 4 ml ($50*10^6$ beads) magnetic beads we used the 24-tip-magnet head and 24 deep-well plates (maximum volume 5 ml). Beads were thoroughly collected when transferred between plates using two collection (30 seconds from the previous plate) and release (5 seconds fast release into next plate) cycles to minimize bead loss. Before collection, the beads were mixed in the previous plate for 1 min at medium speed. The coupling procedure was performed in the dark at room temperature.

1) 4ml of beads (n=$50*10^6$ beads) were transferred from original Luminex flasks to each one well of a 24-deep-well plate. Beads were transferred from plate 1 to plate 2 as described above.
2) <u>Activation 1:</u> Beads were incubated in 3 ml activation buffer (100 mM Na_2HPO4, pH 6.2). The program was paused to add activation reagents to the activation buffer already contained in plate 3 (step 3). After dispensing both reagents manually to plate 3, the beads were mixed for 1 min in plate 2, and then transferred from plate 2 to plate 3.
3) <u>Activation 2:</u> Beads were incubated in activation buffer (1.6 ml) plus EDC (200 µl 1-(3-Dimethylaminopropyl)-3-Ethylcarbodiimide (Pierce 22980) at concentration 50 mg/ml) and NHS (200 µl N-hydroxysuccinimide (Pierce 24500), at concentration 50 mg/ml in waterfree DMSO (Fluka 41647)) for 20 min. Every 2 minutes, a mixing step (30 s, medium speed) was conducted to stir up beads and prevent them from forming a pellet. Afterwards the beads were transferred to plate 4 (see step 4).
4) <u>Coupling buffer 1:</u> Beads were mixed for 1 min (medium speed) in 2 ml coupling buffer (50 mM MES, pH 5.0). Afterwards, the beads were transferred to plate 5 for a second washing step.
5) <u>Coupling buffer 2:</u> Beads were mixed for 1 min (medium speed) in 2 ml coupling buffer, and then transferred to plate 6 (step 6).
6) <u>Binding:</u> Beads were incubated with GC (250 µg/ml) in 2 ml coupling buffer for 2 h. Every 2 minutes, the beads were mixed for 30 s with medium speed. The beads were transferred to plate 7 (step 7)
7) <u>Washing:</u> Beads were washed in 2 ml washing buffer (PBS, 0.05% Tween 20, pH 7.4) and mixed for 1 min (medium speed) before they were transferred to plate 8 (step 8).
8) <u>Storage:</u> Beads were transferred into 2 ml storage buffer (PBS, 1 mg/ml Casein, 0.05% Sodium azide, pH 7.4).

Afterwards, the beads were collected in a manual procedure by rinsing each well (3x 2 ml of storage buffer).

Quality and homogeneity of the automatically coupled magnetic beads were confirmed (Supplementary Figure 2) and compared to a manually coupled non-magnetic bead set. No batch effects or systematic differences compared to the non-magnetic bead set were observed. Coefficients of variation of measured median fluorescence intensities (MFI) per bead set ranged between 4% (375 beads per bead set) and 12% (9000 beads per bead set), and coefficients of variation of bead counts ranged between 9% and 10%. Sufficient bead counts (i.e., >100) were measured for all bead concentrations except 375 beads per set. The quality of the automatically

coupled magnetic beads was further validated using the pathogen-specific reference panels as described in Section 4. Assay validation.

In situ affinity purification of antigens on GC beads

The recombinant bacterial expression of GST-X-Tag fusion antigens and loading of antigens onto beads have been described in detail before[1, 3, 4]. Loading of each antigen onto one magnetic bead set was not automated. In total, 45 bead sets were loaded with pathogen-specific antigens while one bead set was loaded with GST-Tag for background determination and subtraction.

Serum pre-incubation

UKB study sera were shipped to DKFZ on dry ice in a 1:5 serum pre-dilution in preincubation buffer containing Super Chemiblock Heterophile Blocking Agent (Merck Millipore), a proprietary augmenting reagent for removing Rheumatoid Factor and/or HAMA-like interfering antibodies to suppress unspecific signals thereby increasing the signal-to-noise ratio of the serological assay. This reagent pre-absorbs IgM antibodies and strongly reduces IgA antibody signals[5, 6]. On the assay day, sera were further diluted to a serum dilution of 1:500 in the same preincubation buffer at DKFZ and incubated on a shaker at room temperature for one hour.

Multiplex Serology assay

The 46 antigen-loaded bead sets were mixed and 50µl (2500 beads / bead set) were incubated with 50µl of preincubated serum in 96-well filter bottom plates (MSBVN1250, Merck Millipore) yielding a final serum dilution of 1:1000. Washing and incubation steps were performed as previously described[1]. As secondary antibody, a biotinylated goat anti-human IgG/IgM/IgA antibody (109-065-064, Jackson ImmunoResearch) was used in dilution 1:1000. Due to the presence of the Super Chemiblock Blocking Agent, the obtained antibody signals solely reflect IgG levels[5, 6]. As reporter dye, streptavidin-R-phycoerythrin (StrepPE; SAPE-001, MOSS, Inc) was used in dilution 1:750.

UKB samples were analysed using Multiplex Serology on six assay days in two subsequent calendar weeks. In every assay week, antigen loading onto magnetic beads took place on Monday, each of 20 96-well plates (92 samples, 4 plate controls) were tested on Tuesday-Thursday. Plates were measured on the next day (Wednesday-Friday) using two Luminex 200 readers.

Quality controls

At different stages during assay performance, measurements and data processing the following quality controls were included:

1) On each 96-well assay plate, four wells were used for quality controls. Three wells were used for control sera included on every plate to monitor plate handling (discussed in further detail in Section ‘5’ below). In addition, one well per plate was used as blank (no serum) for background subtraction during data processing.
2) To assess and control for assay drift, two plates (n=184 samples) were processed on each assay day within one assay week. To be able to compare assay performance across both assay weeks, 2 plates (n=184 samples) analysed on the first assay day (Tuesday, week I) were also analysed on the first assay day of week II (Tuesday). For further information see section ‘Hardware, software and data processing’.
3) The UKB sera were aliquoted in 96-well plates and further diluted (as described above) by lab personnel at DKFZ using manual multichannel pipettes on assay days. Pipetting errors, also including sera too viscous to ensure pipetting of correct sample volume, were documented. Corresponding sera were later excluded from the final data set and data analysis.
4) For each serum and antigen, at least 100 beads were measured to determine seroreactivity as median fluorescence intensities. When insufficient bead counts occurred during measurements, the corresponding sera were excluded from the final data set.
5) During aliquoting of sera at UKB, lab personnel (independent of DKFZ personnel) interspersed 107 spiked duplicate samples among regular samples. The DKFZ lab and data processing personnel was blinded to the study sample versus spiked duplicate status via pseudonymised IDs, and provided UKB with a complete final data set including the study samples and spiked duplicates. Quality control of the performance of blind spiked duplicates was performed by UKB personnel and included calculation of

coefficient of variations within and between batches (see section 5. UK Biobank Multiplex Serology quality control**)**

Hardware, software and data processing

We used two Luminex 200 devices running with the Luminex XPonent software for measurements of magnetic beads. For each bead set, at least 100 beads / well, i.e. serum were, measured (DD gate: 7000-20,000; 90 seconds sample time out, sample volume 75µl).

Raw data were further processed and analysed using self-implemented scripts (SAS 9.4) for automated background subtraction and assignment of measurements and pseudonymised sample ID. Per antigen (i.e. bead set), mean background reactivity across all plates per assay week were subtracted. Furthermore, one of the 46 included bead set was not loaded with a pathogen-specific GST-X-Tag fusion protein, but only with a GST-Tag fusion protein for determination of serum-specific background (e.g. against *E. coli* proteins, GST-Tag or polystyrene beads). This background was subtracted from all serum-specific antibody measurements. MFI <0 were set to 1 MFI.

During data processing, sera with documented pipetting or measurement errors were excluded. In addition, for each serum the mean seroreactivity was calculated. In cases where no or insufficient pipetting of sample was suspected, measurement data were checked.

As described above, on each assay day, 184 samples, i.e. bridging panels, were re-measured to assess and account for potential batch effects (inter-assay-day, inter-assay-week. The sero-reactivity in each of these pairwise sets of bridging panels were assessed by scatterplots (slope, correlation) and seroprevalences, and correction factors were calculated.

Quality control analyses were performed using Microsoft Excel 2016. For graphical visualisation, R (version number 3.3.1, June 2016) and Microsoft Excel 2016 were used.

4. *Assay validation*

We have adopted the terms “monoplex” (an assay with one or multiple antigen:bead sets all relating to a single infectious agent), and “multiplex” (where multiple antigen:bead sets were present in the same reaction relating to multiple infectious agents) in our assay validation approach to address possible concerns related to assay inter-relatedness.

The validation was performed in a stepwise approach as described in Supplementary Figure 1. First, individual pathogen-specific Monoplex Serology assays were compared to established reference assays based on non-magnetic beads[7, 8, 9, 10]. Second, based on non-magnetic beads, assay performance of the pathogen-specific assays in both monoplex and multiplex format, i.e. comprising the full UKB Multiplex Serology antigen panel, was performed[4, 8, 9, 11]. The first and second validation steps have been reported previously and are summarised in Supplementary Table 2. In the third step, pathogen-specific assay performance in multiplex format was validated by comparing their performance on non-magnetic versus magnetic beads. To compare pathogen-specific assay performance on non-magnetic and magnetic beads in multiplex format, sensitivity, specificity and Cohen’s *kappa* were calculated in comparison to the reference assay used in Monoplex Serology validation[7, 8, 9, 10] (Supplementary Figure 3). For all pathogen-specific Multiplex Serology assays, similar performance characteristics were observed on non-magnetic and magnetic beads. To further assess agreement of Multiplex Serology on non-magnetic versus magnetic beads in determining seropositivity, Intra-class Correlation Coefficients (ICCs)[12] were calculated using the package ‘psych’ in R 3.5.0. ICCs directly comparing the performance of Multiplex Serology on magnetic vs. non-magnetic beads were above 0.8 indicating good to excellent reliability, except for Tg where the ICC was 0.48 (Supplementary Table 3). This was likely due to lower specificity but higher sensitivity on non-magnetic versus magnetic beads (Supplementary Figure 2).

A possible concern in multiplex serology assay development is the potential for cross-reactivity between antigen:bead sets in the same reaction, i.e. different intensities measured for the same sample depending on whether the reaction was performed in a monoplex or multiplex format. If that was true, one would expect to observe an increased background in a multiplexed reaction that would alter the number of samples being classified as seropositive or negative. Since we had access to multiple sera with defined ‘true’ status through using reference assays we used these sera to determine whether the sensitivity and specificity metrics estimated in the monoplex assays were affected by multiplexing[7, 8] The only agents found to have significant discrepancies in estimated metrics between monoplex and multiplex were *Toxoplasma gondii* (*Tg*) and human T-lymphotropic virus-1 (HTLV-1). For *Tg*, these differences were likely observed owing to insufficient volumes remaining for

12% of reference sera, i.e. different numbers of sera tested monoplex versus multiplex validation. Furthermore, lower sensitivity of the multiplex assay was expected for *Tg* given the low signal intensities observed in earlier validation work (at 1:100 serum dilution) and the increased dilution used in the UKB multiplex panel (1:1000)[8]. For HTLV-1, an increase in the background for the Gag antigen was observed for unknown reasons. This was the only antigen which required a significant increase in cut-off to determine seropositivity in the multiplex assay.

5. *UK Biobank Multiplex Serology quality control*

The specific methodology used to test the serum samples from 9,695 individuals from UKB is described in the main text, and Supplementary Materials Section 4. Of the total 10,110 serum samples assayed, 29 samples (0.3%) were excluded from analyses (2 highly viscous sera, 8 pipetting errors, 8 were incorrectly diluted and 11 with insufficient bead counts at the reading step). The remaining samples represented either study participants (n=9,695), blind-spiked duplicates (n=107) or repeat assessment encounter samples (n=277) or samples initially destined for repeat assessment samples invalidated due to other errors (n=2). After exclusion of invalid samples (n=29), we observed little evidence of batch effects as demonstrated by comparing antigen-specific MFI values for duplicate samples measured across 2 weeks (Supplementary Figure 4), with a summary of all comparisons shown in Supplementary Table 4. Coefficients of variation (CV) measured for blind spike duplicates for 107 individuals were calculated using the logarithmic method of Bland and Altman[11]. CV was calculated both for all individuals, and among seropositives only. It was hypothesised that the CV calculations including all samples in those infectious agents less prevalent in the population would be more likely to be subject to more random noise, especially those with a median reactivity below the lower limit of quantitation (approx. 30 MFI at 1:1000 serum dilution). Among all samples, CVs ranged between 7.8% (*Hp* CagA) and 35.4% (*Hp* GroEL) with a median across all antigens of 17%. Among seropositives only (not available for the HCV and HTLV-1 antigens, as well as HIV-1 Env and HPV-16 E6), CVs ranged between 0.2% (*Ct* PorB) and 12.0% (*Ct* pGP3) with median of 3.5% (Supplementary Table 5).

In addition, control sera were included on each assay plate to monitor individual plate handling by assessing inter-plate variance. As an example, seroreactivity for one of the control sera for all antigens and all plates is shown in Supplementary Figure 5. Median intra-day CVs ranged between 11% and 23% (median 16%), median intra-week CVs ranged between 12% and 22% (median 19%) and CVs across all assay days ranged between 16% and 26% (median 21%).

Samples for repeat assessments were available for 277 individuals tested in a blinded fashion. Seroconversion rates within the 3-5 year interval between baseline and repeat assessments ranged from 0%-10.5% but seroreversion rates of 0-9.4% were also observed (Supplementary Table 6). For those infectious agents where a single antigen was used to define seroprevalence, over 70% of samples demonstrating discordant results between repeat samples had MFI values clustering within a single standard deviation around the cut-off for each antigen. Thus, these mismatches are likely a result of the CV of the assay and the remaining 10-30% of discordant samples are likely to represent true seroconverters or reverters. For one measured antigen (*Hp* CagA) bead-antigen complexing was accidentally omitted in the second week, therefore sero-reactivity and sero-positivity estimates for this infectious agent were derived for only 50% of the tested samples.

6. *Multiplex Serology seroprevalence calculations from antigen reactivity data*

The final 45 antigens tested in the UKB panel are listed in Supplementary Table 2. The interpretation of the individual antigen positivity data to calculate seroprevalence estimates for each agent was as follows:

Herpes simplex virus 1 (HSV-1): Reactivities against gG (1gG; cut-off: 150 MFI) antigen alone were used for prevalence estimates[7].

Herpes simplex virus 2 (HSV-2): Reactivities against mgGunique (2mG; cut-off: 150 MFI) antigen alone were used for prevalence estimates[7].

Varicella zoster virus (VZV): gE and gI antigens were co-bound to a single bead set and the combined reactivity (cut-off: 100 MFI) was used to calculate seroprevalence estimates[7].

Epstein Barr virus (EBV): Seropositivity was defined if individuals were seropositive against at least two antigens from a total of VCAp18 (cut-off: 250 MFI), EBNA1 peptide (cut-off: 250 MFI), ZEBRA (cut-off: 100 MFI) and EA-D (cut-off: 100 MFI)[7].

Cytomegalovirus (CMV): Seropositivity was defined if individuals were sero-reactive for at least 2 antigens from a total of pp150N (cut-off: 100 MFI), pp52 (cut-off: 150 MFI) and pp28 (cut-off: 200 MFI)[7].

Human herpes virus 6 (HHV-6): Sero-positivity was defined if seropositive against any of the three tested HHV-6 antigens (IE1A cut-off: 100 MFI, IE1B cut-off: 100 MFI, p101k cut-off: 100 MFI). Species-specific seropositivity for HHV-6A and B were defined if seropositive for IE1A or IE1B, respectively. However, as no reference assay was available for HHV-6, these classifications should be considered preliminary.

Human herpes virus 7 (HHV-7): Reactivities against U14 antigen alone were used for prevalence estimates (cut-off: 100 MFI). However, as no reference assay was available for HHV-7, this classification should be considered preliminary. Traditionally, there have been concerns of cross-reactivity between HHV-6 and HHV-7 species antibody responses. We tested for evidence of cross-reactivity and found very little correlation between magnitude of responses against HHV-6 and HHV-7 antigens in our UKB dataset (r<0.2 for all inter-agent comparisons; Supplementary Figure 6).

Kaposi's sarcoma-associated herpesvirus (KSHV): Seropositivity was defined in individuals seropositive against LANA (cut-off: 100 MFI) AND/OR K8.1 (cut-off: 175 MFI) antigens.

Hepatitis B virus (HBV): Seropositivity was defined in individuals seropositive against HBc (cut-off: 100 MFI) AND HBe (cut-off: 150 MFI) antigens[8]. HB_S antigen was not included and thus measures are likely to represent an unbiased picture of HBV exposure rather than being confounded by vaccination.

Hepatitis C virus (HCV): Seropositivity was defined in individuals seropositive against Core (cut-off: 150 MFI) AND NS3 (cut-off: 150 MFI) antigens[8].

HTLV-1: Seropositivity was defined in individuals seropositive against Gag (cut-off: 1500 MFI) AND/OR Env (cut-off: 150 MFI) antigens. Increased reactivities were observed for HTLV-1 Gag responses that could be accounted for by increasing the cut-off[8].

Human immunodeficiency virus 1 (HIV-1): Seropositivity was defined in individuals seropositive against Gag (cut-off: 600 MFI) AND Env (cut-off: 150 MFI) antigens[9]

Human papillomavirus 16 (HPV-16): Reactivities against L1 antigen (cut-off: 175 MFI) alone were used for prevalence estimates[1, 13]. Antibodies against HPV-16 oncoproteins E6 (cut-off: 120 MFI) and E7 (cut-off: 150 MFI) are markers of prevalent or incident HPV-16-driven anogenital or oropharyngeal cancer[14, 15, 16]. Seropositivity to HPV-16 E6 is a biomarker for future development of HPV-16-driven oropharyngeal cancer[17, 18, 19].

Human papillomavirus 18 (HPV-18): Reactivities against L1 antigen (cut-off: 175 MFI) alone were used for prevalence estimates[1, 4, 13].

JC virus (JCV): Reactivities against VP1 antigen (cut-off: 250 MFI) alone were used for prevalence estimates[20, 21, 22].

BK virus (BKV): Reactivities against VP antigen (cut-off: 250 MFI) alone were used for prevalence estimates[20, 21, 22].

Merkel cell virus (MCV): Reactivities against VP1 antigen (cut-off: 250 MFI) alone were used for prevalence estimates[20, 21, 22].

Chlamydia trachomatis (*Ct*): Reactivities against pGP3 antigen alone (cut-off 200 MFI) were used for prevalence estimates[10].

Hp: Seropositivity was defined if individuals were seropositive against at least two antigens from a total of CagA (cut-off: 400 MFI), VacA (cut-off: 100 MFI), OMP (cut-off: 170 MFI), GroEL (cut-off: 80 MFI), Catalase (cut-off: 180 MFI) and Urease (cut-off: 130 MFI). This classification was only possible for the samples tested in the first week (n=4,871) as discussed above. Therefore all estimates for *Hp* are only provided for the samples tested in the first week.

Tg: Seropositivity was defined in individuals being seropositive against sag1 (cut-off: 160 MFI) AND/OR p22 (cut-off: 100 MFI) antigens[8].

For all infectious agents, the calculated seroprevalence estimates were compared to those in the general literature using a search method on PubMed undertaken using the following terms using CMV as an example:

(((cytomegalovirus[Title/Abstract] OR CMV[Title/Abstract]) AND (seroprevalence[Title/Abstract] OR IgG[Title/Abstract] OR antibody[Title/Abstract] OR prevalence[Title/Abstract]) AND (united kingdom[Title/Abstract] OR UK[Title/Abstract]OR europe[Title/Abstract])))

Only studies with more than 500 individuals were considered and studies were excluded if they primarily described individuals with a diagnosis of HIV-1 (except in the case of estimating HIV-1 seroprevalence), prior solid organ or bone marrow transplantation, or those receiving haemodialysis. Reported seroprevalence estimates were prioritised for UK or European adult populations between 40-70 years of age, but other populations were considered if no data from European studies could be identified.

7. UK Biobank Data

Data was collected from UKB participants using a variety of methods and at separate encounters. Only defined phenotypes thought to be linked with infectious disease exposures or outcomes were curated and tested for association with tested antibody traits. These included:

Age: Date of birth collected from NHS central registries and confirmed by the participant at the questionnaire stage of baseline recruitment (Field 33). Age was calculated by subtracting date of birth from the date of first attendance (Field 53).

Sex: Collected from NHS central registries and confirmed by the participant at the questionnaire stage of baseline recruitment (Field 31).

Ethnic Group: Collected during touch-screen questionnaire at baseline recruitment encounter (Field 21000). Individuals were divided into categories of 'White', 'Asian', 'Black', or 'Other' (if not one of the other three) based on self-report.

Townsend deprivation index (TDI): Calculated immediately prior to participant joining UK Biobank. Based on data from the preceding national census and the participant's postcode area at the time of invitation to join. Since the data was positively skewed, individuals were divided into quintiles (Field 189).

Household size: Collected during touch-screen questionnaire at baseline recruitment encounter as 'number in household' question (Field 709). Individuals were clustered into categories of 'Lives Alone', '2', '3', '4', '5 or More'.

Tobacco smoking status: Collected during touch-screen questionnaire at baseline recruitment encounter as 'smoking status' question (Field 20116) classifying individuals as a 'Current', 'Previous' or 'Never' smoker.

Alcohol drinking status: Collected during touch-screen questionnaire at baseline recruitment encounter as 'alcohol drinker status' question (Field 20117) classifying individuals as 'Current', 'Previous' or a 'Never' smoker.

Reported number of lifetime sexual partners (LSP): Collected during touch-screen questionnaire at baseline recruitment encounter as 'lifetime number of sexual partners' question (Field 2149). This includes vaginal, anal and oral sex partners. Individuals were classified into groups of lifetime partners including: '0', '1', '2-4', '5-10', 'Greater than 10'.

Same-sex intercourse ever (sameSI): Collected during touch-screen questionnaire at baseline recruitment encounter as 'ever had same-sex intercourse' (Field 2159). Individuals were classified into groups dependent on whether they answered 'yes' or 'no' to this question.

Disease case: Defined using self-report of diagnoses collected during touch-screen questionnaire (Field 20002) at any patient encounter for multiple sclerosis (MS; code 1261) or coeliac disease (code 1456). Cases of cervical cancer and CIN were defined using ICD9 (2331 and 1808; and 1809 respectively), and ICD10 (D06, D06.1, D06.7; and C53 respectively) codes derived from National Health Service cancer registry data. ICD9 codes were derived from Field 40013 and ICD10 in Field 40006.

8. *Testing for association between demographic variables and infectious agent serostatus*

Features such as sex, age, reported ethnicity, number of individuals per household, social deprivation status and lifetime number of sexual partners have been associated with risk of infection with a number of agents. We therefore set out to test associations between such factors as measured in UKB and infectious agent serostatus using a step-wise approach. Firstly, univariate logistic regression models testing association between the measured exposure variable and seropositivity were compared to a null model using the likelihood ratio test. Any comparisons that were found to be significant using this approach ($P<0.05$) were then taken forwards for testing in a multivariable logistic model including other measured variables as covariates. After inclusion of all other measured variables, no infectious agent serostatus demonstrated any significant association either alone or in a trend with the number of individuals in a household therefore this was not analysed further. Supplementary Tables 7-12 provide statistics of association between antigen or infectious agent seroprevalence and demographic factors including sex, age, ethnicity, TDI and LSP as detailed further in the main text.

9. *Genetic association tests in UKB*

Data from direct genotyping and imputation release 3 of UKB data were used in the genetic analyses. Only individuals and SNPs passing basic quality control (QC) steps (missingness or heterozygosity in any batch) were included in the final genetic analyses[23]. Of the 9,695 individuals with demographic and antibody data, 9,611 individuals had genetic data available that was of sufficient quality for analysis after QC. Three genetic variants were tested for association with a variety of traits as described in Supplementary Table 12. Two of these variants were imputed for which we assessed INFO scores as measures of imputation accuracy.

The genetic data was used for three purposes. The first was to attempt to replicate associations between three genetic variants in the major histocompatibility complex reported with JCV and MCV serostatus, and EBV EBNA1 magnitude of antibody response (described as a continuous variable). We tested only variants reported in a single publication performed in a population of European ancestry using association statistics available from Table 2 within that particular manuscript[24]. Since the vast majority of individuals in our UKB dataset were of reported British or European ancestry we used all individuals with genetic data for this analysis. To account for other individuals of non-European ancestry and to account for cryptic relatedness we used two linear mixed-model association testing software systems. To test association with directly genotyped variants we used GCTA (v1.26.0)[25]. This software calculates a genetic relatedness matrix and includes this matrix as a random effect covariate. We used the entire post-QC dataset of 734,447 variants across the autosomes in addition to age (in years) and sex as fixed effect covariates. For imputed variants we used BOLT-LMM (v2.3.1) to account for imputation probabilities that calculates an estimate of the genetic relatedness matrix from the genotyped variants[26]. In both cases only beta values and standard errors were calculated even if the trait was binary and therefore odds ratios were calculated for UKB traits that were tested in a binary format using the Shiny app (http://cnsgenomics.com/shiny/LMOR/).

Secondly, we tested for association with all imputed variants and $\log_{10}$ normalised quantitative responses against EBV VCAp18 using BOLT-LMM (v2.3.1) including age and sex as fixed effect covariates. This antigen was selected as a marker of ever being exposed to the viral capsid protein of EBV rather than a marker of replication that, alone, may be less sensitive. The resultant Manhattan plot from these autosome-wide associations is included in Supplementary Figure 5.

The third set of analyses involved testing for genetic correlation between antibody response traits and disease outcomes (MS and coeliac disease). Firstly, association statistics for HLA alleles were calculated using a large independently collected case-control dataset of MS (International MS case-control) using only data across the MHC region[27]. For the International MS dataset including 17,610 cases and 30,129 controls, genotyped variants across the MHC were used to impute HLA alleles using SNP2HLA (vv1.0.2)[28] and the Type I Diabetes Genomics Consortium reference dataset. The resultant ‘hard’ called alleles were then tested for association between cases of MS and matched population controls within each individual population using logistic regression in PLINK (v1.9)[29]. The statistics from each population were then combined using a fixed effects meta-analysis in METASOFT (v2.0.1)[30]. These statistics were compared to those equivalent for imputed HLA alleles within the UKB dataset. HLA alleles were imputed for the 9,611 individuals with genotype and antibody data available again using UKB genotype data using SNP2HLA and the T1DGC reference dataset. The alleles were tested for association with quantitative EBNA1 and VCAp18 levels using GCTA with the GRM calculated using genotypes across all autosomes and controlling for sex and age. To formally test for genetic correlation between traits we used a refined dataset of 6,265 unrelated individuals of strict European descent in the UKB subset described here to determine association estimates against antibody responses. Then, using the bivariate GREML analysis toolset in GCTA (v1.26.0)[31] we compared the association of genotyped variants alone against 11,236 individuals of unrelated European descent selected from the remainder of the complete UKB dataset

(1,236 cases of MS and 10,000 random controls with no record of MS). This analysis was undertaken using either all autosomal data or only variants across the MHC. The same analysis was undertaken for coeliac disease using an additional total of 11,468 individuals (1,468 cases and 10,000 additional randomly selected European controls).

10. Supplementary Figures

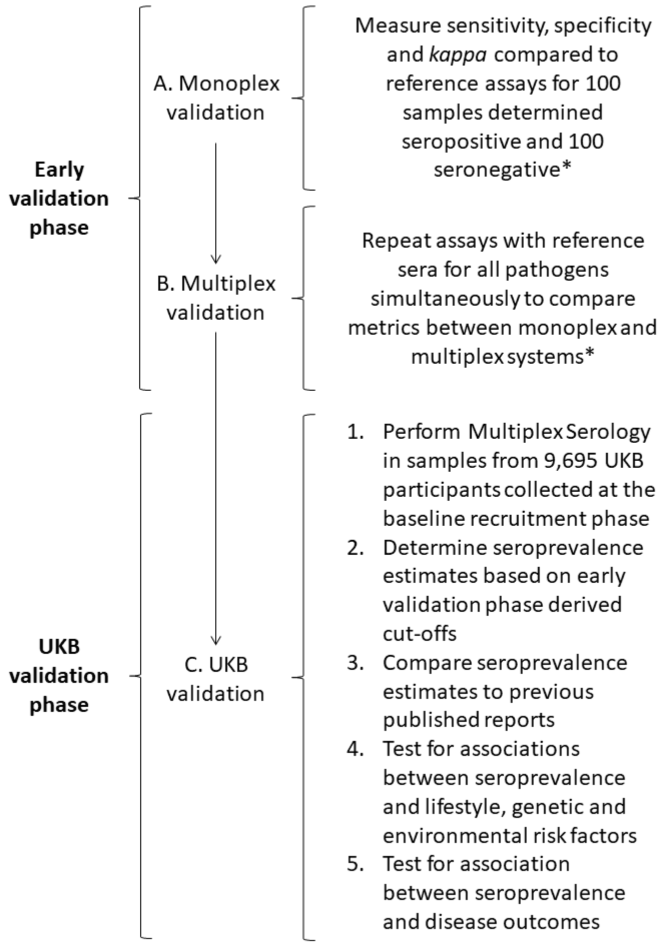

Supplementary Figure 1: Proposed steps of validation for infectious agents of highlighted relevance to public health.
A) The Multiplex Serology assay for each infectious agent was tested individually (i.e. 'Monoplex') against reference sera defined as positive or negative using established reference assays to estimate performance characteristics. B) All assays were performed simultaneously in a multiplex reaction using the reference sera available from step A) to determine whether performance characteristics were adversely affected by multiplexing. This step was performed on non-magnetic and magnetic beads. C) The Multiplex Serology platform was used for 9,695 samples available from UK Biobank to estimate seroprevalence of the multiple infectious agents across the UK using cut-offs defined in the early validation phase. Simple associations between seroprevalence and lifestyle, environmental and genetic risk factors were then tested using the refined cut-offs.
*: Reference sera were available for 11 out of the total 20 infectious agents included in the panel.
kappa: Cohen's *kappa*.

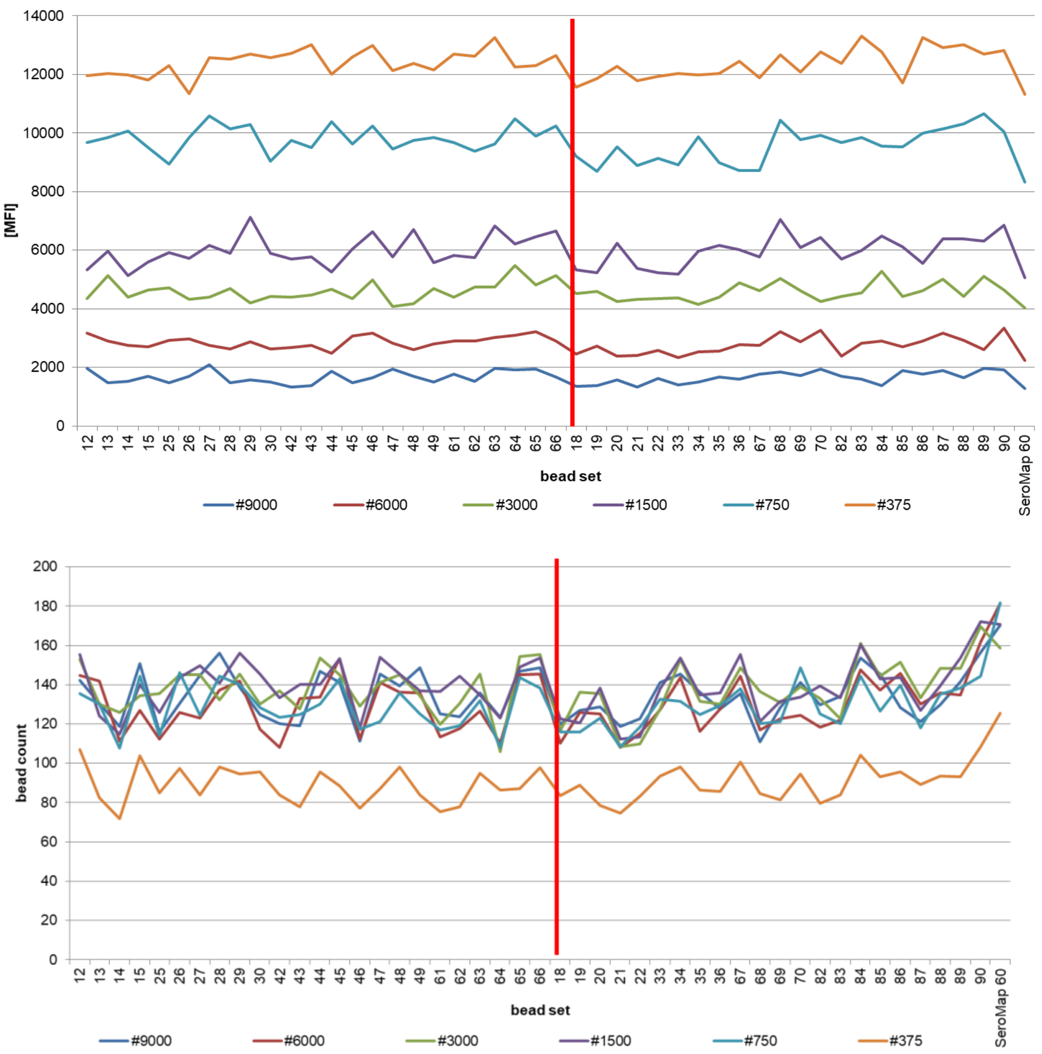

Supplementary Figure 2: Quality control of automatically GC-coupled magnetic bead sets.

The 46 automatically coupled bead sets (from left to right, bead set 12 to bead set 90) were compared to a manually coupled non-magnetic bead set (SeroMap bead set 60, rightmost). Per bead set, 375 beads (orange line) to9000 beads (dark blue line) were loaded with recombinantly expressed GST-Tag protein, and incubated with a biotinylated mouse monoclonal antibody directed against the Tag. Bound GST-Tag protein was detected using StrepPE and quantified using a Luminex 200 reader. The red line divides the 46 bead sets into the coupling batches I and II. Upper panel: Measured median fluorescence intensities (MFI) per bead set, indicating homogeneous coupling of GC to the 46 bead sets. Coefficients of variation in reactivity ranged between 4% (375 beads per bead set) and 12% (9000 beads per bead set). Lower panel: Measured bead counts, indicating sufficient bead counts (i.e., >100) for all bead concentrations except 375 beads per set. Coefficients of variation of bead counts ranged between 9% and 10%.

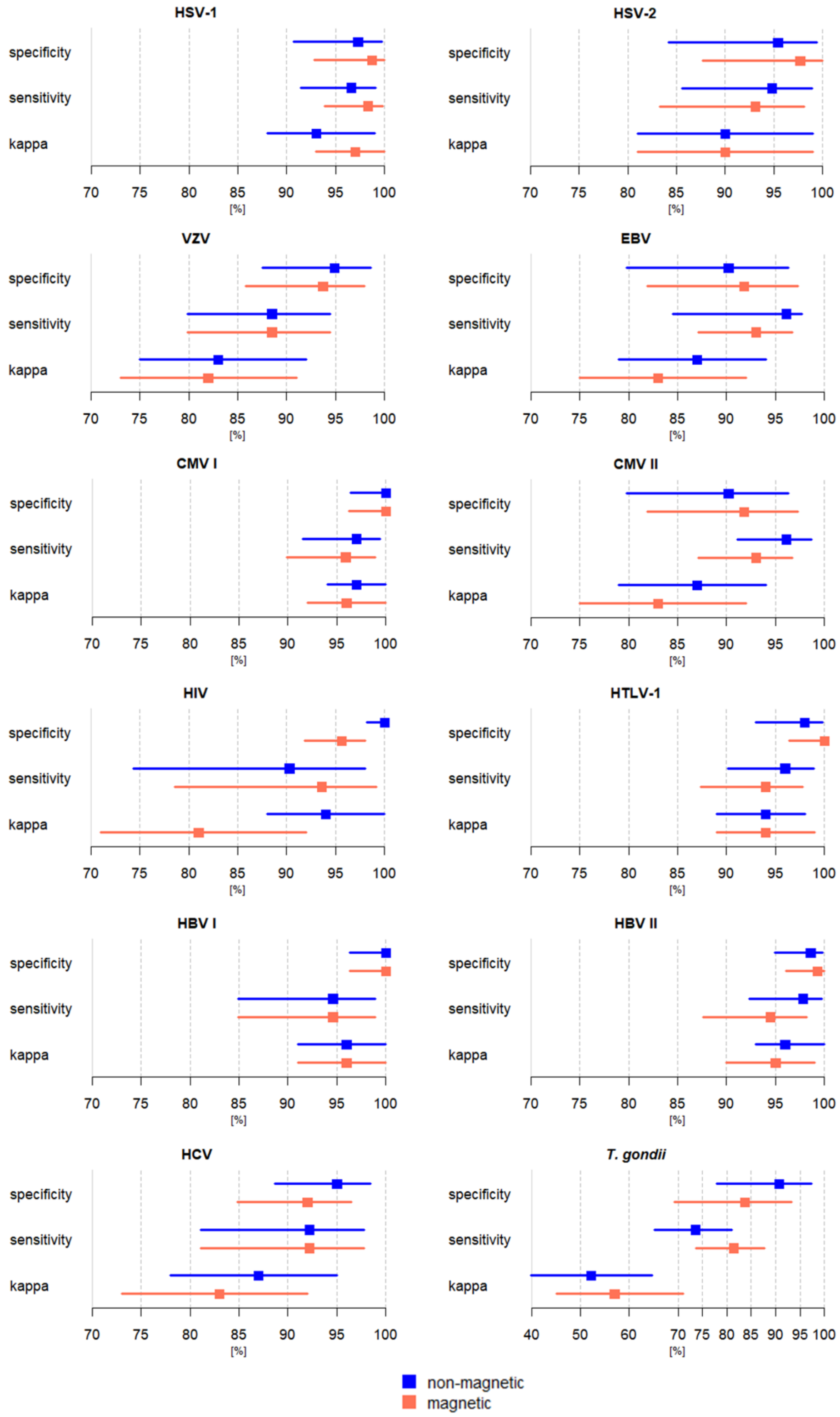
HSV-1
HSV-2
VZV
EBV
CMV I
CMV II
HIV
HTLV-1
HBV I
HBV II
HCV
T. gondii
specificity
sensitivity
kappa
70 75 80 85 90 95 100
[%]
40 50 60 70 75 80 85 90 95 100
non-magnetic
magnetic

Supplementary Figure 3: Comparison of statistical characteristics (specificity, sensitivity and Cohen's kappa) in Multiplex Serology on non-magnetic (blue) vs. magnetic (orange) beads for 10 infectious agents.
Multiplex Serology on non-magnetic beads was previously validated against reference assays based on designated reference sera (from n=203 (HSV-1), n=107 (HSV-2), n=180 (VZV), n=201 (EBV), n=205 (CMV I), n=201 (CMV II), n=244 (HIV), n=200 (HTLV-1), n=157 (HBV I), n=220 (HBV II), n=154 (HCV), n=198 (*T. gondii*) biologically independent samples described in Brenner et al. 2018, 2019[7, 8, 9]). In case of CMV and HBV, two sets of reference sera originally tested using two different reference assays each were available as indicated by roman numbers. Reference sera were re-tested with Multiplex Serology using magnetic beads and statistical performance against the corresponding reference assay was re-evaluated. Error bars indicate 95% confidence intervals. Magnetic beads were used for the final UKB Multiplex Serology platform.

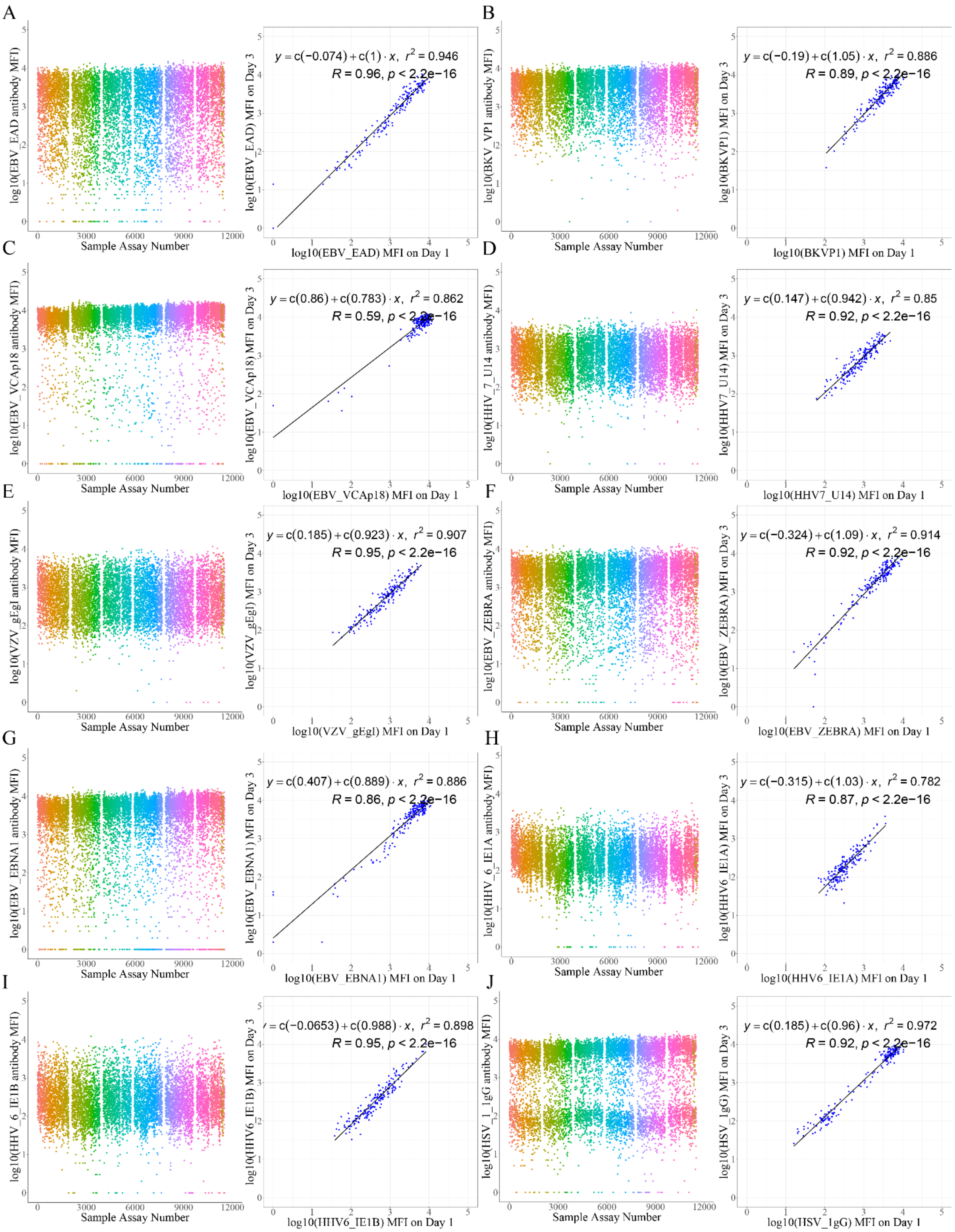
A
log10(EBV_EAD antibody MFI)
Sample Assay Number
y = c(−0.074) + c(1) · x, r² = 0.946
R = 0.96, p < 2.2e−16
log10(EBV_EAD) MFI on Day 3
log10(EBV_EAD) MFI on Day 1
B
log10(BKV_VP1 antibody MFI)
Sample Assay Number
y = c(−0.19) + c(1.05) · x, r² = 0.886
R = 0.89, p < 2.2e−16
log10(BKVP1) MFI on Day 3
log10(BKVP1) MFI on Day 1
C
log10(EBV_VCAp18 antibody MFI)
Sample Assay Number
y = c(0.86) + c(0.783) · x, r² = 0.862
R = 0.59, p < 2.2e−16
log10(EBV_VCAp18) MFI on Day 3
log10(EBV_VCAp18) MFI on Day 1
D
log10(HHV_7_U14 antibody MFI)
Sample Assay Number
y = c(0.147) + c(0.942) · x, r² = 0.85
R = 0.92, p < 2.2e−16
log10(HHV7_U14) MFI on Day 3
log10(HHV7_U14) MFI on Day 1
E
log10(VZV_gEgI antibody MFI)
Sample Assay Number
y = c(0.185) + c(0.923) · x, r² = 0.907
R = 0.95, p < 2.2e−16
log10(VZV_gEgI) MFI on Day 3
log10(VZV_gEgI) MFI on Day 1
F
log10(EBV_ZEBRA antibody MFI)
Sample Assay Number
y = c(−0.324) + c(1.09) · x, r² = 0.914
R = 0.92, p < 2.2e−16
log10(EBV_ZEBRA) MFI on Day 3
log10(EBV_ZEBRA) MFI on Day 1
G
log10(EBV_EBNA1 antibody MFI)
Sample Assay Number
y = c(0.407) + c(0.889) · x, r² = 0.886
R = 0.86, p < 2.2e−16
log10(EBV_EBNA1) MFI on Day 3
log10(EBV_EBNA1) MFI on Day 1
H
log10(HHV_6_IE1A antibody MFI)
Sample Assay Number
y = c(−0.315) + c(1.03) · x, r² = 0.782
R = 0.87, p < 2.2e−16
log10(HHV6_IE1A) MFI on Day 3
log10(HHV6_IE1A) MFI on Day 1
I
log10(HHV_6_IE1B antibody MFI)
Sample Assay Number
y = c(−0.0653) + c(0.988) · x, r² = 0.898
R = 0.95, p < 2.2e−16
log10(HHV6_IE1B) MFI on Day 3
log10(HHV6_IE1B) MFI on Day 1
J
log10(HSV_1_IgG antibody MFI)
Sample Assay Number
y = c(0.185) + c(0.96) · x, r² = 0.972
R = 0.92, p < 2.2e−16
log10(HSV_1gG) MFI on Day 3
log10(HSV_1gG) MFI on Day 1

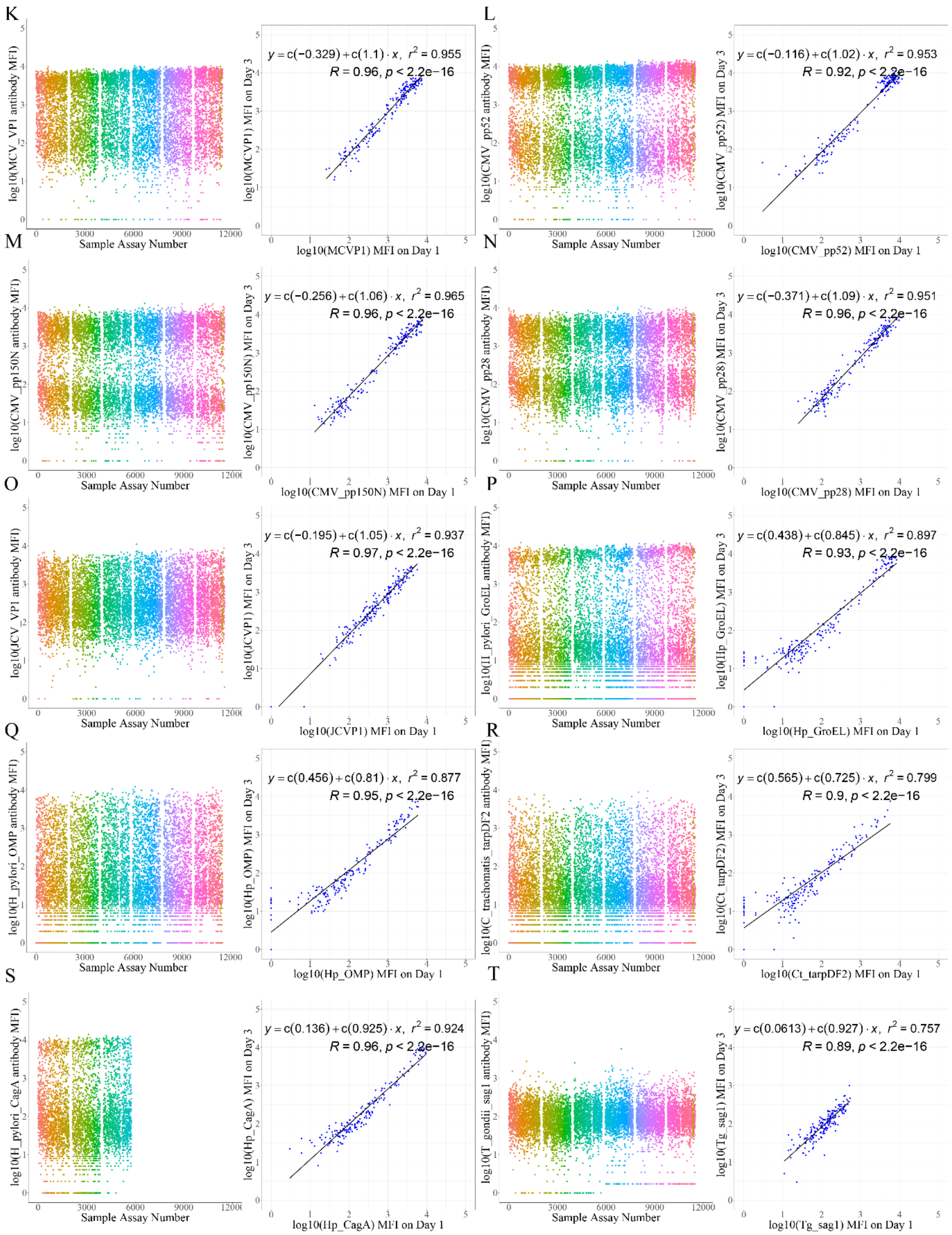
K
log10(MCV_VP1 antibody MFI)
Sample Assay Number
y = c(−0.329) + c(1.1) · x, r2 = 0.955
R = 0.96, p < 2.2e−16
log10(MCVP1) MFI on Day 3
log10(MCVP1) MFI on Day 1
L
log10(CMV_pp52 antibody MFI)
Sample Assay Number
y = c(−0.116) + c(1.02) · x, r2 = 0.953
R = 0.92, p < 2.2e−16
log10(CMV_pp52) MFI on Day 3
log10(CMV_pp52) MFI on Day 1
M
log10(CMV_pp150N antibody MFI)
Sample Assay Number
y = c(−0.256) + c(1.06) · x, r2 = 0.965
R = 0.96, p < 2.2e−16
log10(CMV_pp150N) MFI on Day 3
log10(CMV_pp150N) MFI on Day 1
N
log10(CMV_pp28 antibody MFI)
Sample Assay Number
y = c(−0.371) + c(1.09) · x, r2 = 0.951
R = 0.96, p < 2.2e−16
log10(CMV_pp28) MFI on Day 3
log10(CMV_pp28) MFI on Day 1
O
log10(JCV_VP1 antibody MFI)
Sample Assay Number
y = c(−0.195) + c(1.05) · x, r2 = 0.937
R = 0.97, p < 2.2e−16
log10(JCVP1) MFI on Day 3
log10(JCVP1) MFI on Day 1
P
log10(H_pylori_GroEL antibody MFI)
Sample Assay Number
y = c(0.438) + c(0.845) · x, r2 = 0.897
R = 0.93, p < 2.2e−16
log10(Hp_GroEL) MFI on Day 3
log10(Hp_GroEL) MFI on Day 1
Q
log10(H_pylori_OMP antibody MFI)
Sample Assay Number
y = c(0.456) + c(0.81) · x, r2 = 0.877
R = 0.95, p < 2.2e−16
log10(Hp_OMP) MFI on Day 3
log10(Hp_OMP) MFI on Day 1
R
log10(C_trachomatis_tarpDF2 antibody MFI)
Sample Assay Number
y = c(0.565) + c(0.725) · x, r2 = 0.799
R = 0.9, p < 2.2e−16
log10(Ct_tarpDF2) MFI on Day 3
log10(Ct_tarpDF2) MFI on Day 1
S
log10(H_pylori_CagA antibody MFI)
Sample Assay Number
y = c(0.136) + c(0.925) · x, r2 = 0.924
R = 0.96, p < 2.2e−16
log10(Hp_CagA) MFI on Day 3
log10(Hp_CagA) MFI on Day 1
T
log10(T_gondii_sag1 antibody MFI)
Sample Assay Number
y = c(0.0613) + c(0.927) · x, r2 = 0.757
R = 0.89, p < 2.2e−16
log10(Tg_sag1) MFI on Day 3
log10(Tg_sag1) MFI on Day 1

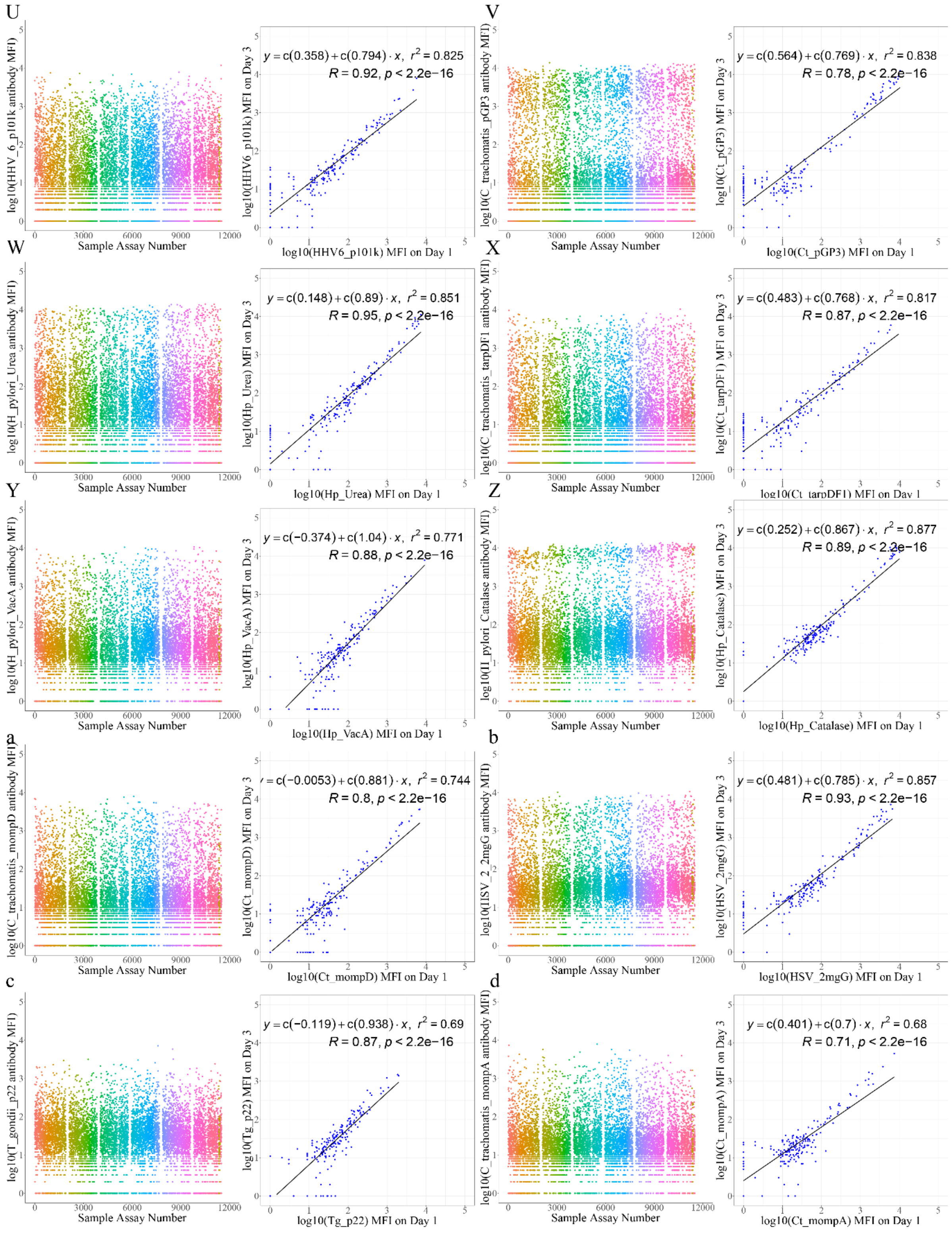
U
V
W
X
Y
Z
a
b
c
d
Sample Assay Number
log10(HHV_6_p101k antibody MFI)
log10(HHV6_p101k) MFI on Day 3
log10(HHV6_p101k) MFI on Day 1
y = c(0.358) + c(0.794) · x, r2 = 0.825
R = 0.92, p < 2.2e−16
log10(C_trachomatis_pGP3 antibody MFI)
log10(Ct_pGP3) MFI on Day 3
log10(Ct_pGP3) MFI on Day 1
y = c(0.564) + c(0.769) · x, r2 = 0.838
R = 0.78, p < 2.2e−16
log10(H_pylori_Urea antibody MFI)
log10(Hp_Urea) MFI on Day 3
log10(Hp_Urea) MFI on Day 1
y = c(0.148) + c(0.89) · x, r2 = 0.851
R = 0.95, p < 2.2e−16
log10(C_trachomatis_tarpDF1 antibody MFI)
log10(Ct_tarpDF1) MFI on Day 3
y = c(0.483) + c(0.768) · x, r2 = 0.817
R = 0.87, p < 2.2e−16
log10(H_pylori_VacA antibody MFI)
log10(Hp_VacA) MFI on Day 3
log10(Hp_VacA) MFI on Day 1
y = c(−0.374) + c(1.04) · x, r2 = 0.771
R = 0.88, p < 2.2e−16
log10(H_pylori_Catalase antibody MFI)
log10(Hp_Catalase) MFI on Day 3
log10(Hp_Catalase) MFI on Day 1
y = c(0.252) + c(0.867) · x, r2 = 0.877
R = 0.89, p < 2.2e−16
log10(C_trachomatis_mompD antibody MFI)
log10(Ct_mompD) MFI on Day 3
log10(Ct_mompD) MFI on Day 1
y = c(−0.0053) + c(0.881) · x, r2 = 0.744
R = 0.8, p < 2.2e−16
log10(HSV_2_2mgG antibody MFI)
log10(HSV_2mgG) MFI on Day 3
log10(HSV_2mgG) MFI on Day 1
y = c(0.481) + c(0.785) · x, r2 = 0.857
R = 0.93, p < 2.2e−16
log10(T_gondii_p22 antibody MFI)
log10(Tg_p22) MFI on Day 3
log10(Tg_p22) MFI on Day 1
y = c(−0.119) + c(0.938) · x, r2 = 0.69
R = 0.87, p < 2.2e−16
log10(C_trachomatis_mompA antibody MFI)
log10(Ct_mompA) MFI on Day 3
log10(Ct_mompA) MFI on Day 1
y = c(0.401) + c(0.7) · x, r2 = 0.68
R = 0.71, p < 2.2e−16

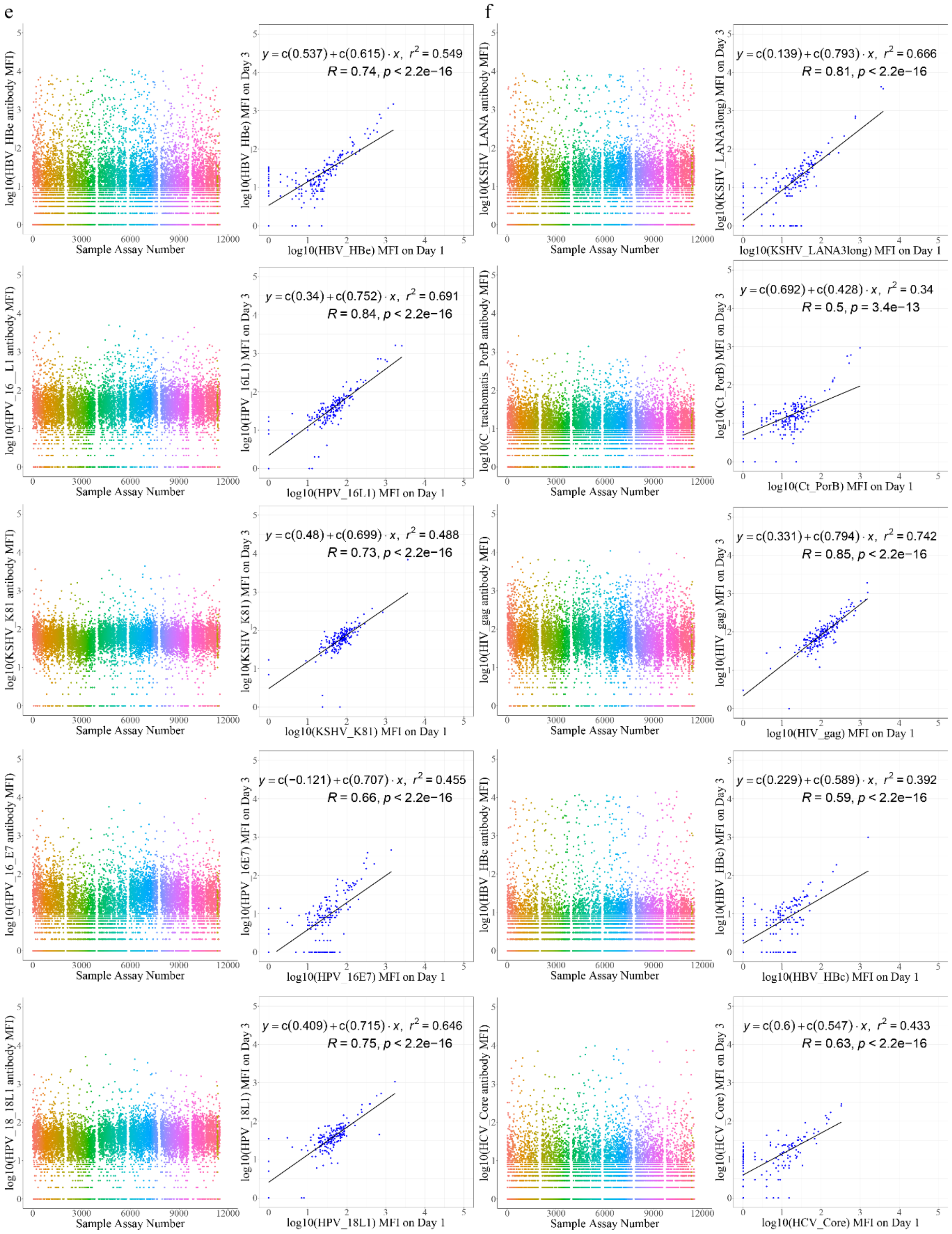
e
f
log10(HBV_HBe antibody MFI)
Sample Assay Number
y = c(0.537) + c(0.615) · x, r² = 0.549
R = 0.74, p < 2.2e−16
log10(HBV_HBe) MFI on Day 3
log10(HBV_HBe) MFI on Day 1
log10(KSHV_LANA antibody MFI)
y = c(0.139) + c(0.793) · x, r² = 0.666
R = 0.81, p < 2.2e−16
log10(KSHV_LANA3long) MFI on Day 3
log10(KSHV_LANA3long) MFI on Day 1
log10(HPV_16_L1 antibody MFI)
y = c(0.34) + c(0.752) · x, r² = 0.691
R = 0.84, p < 2.2e−16
log10(HPV_16L1) MFI on Day 3
log10(HPV_16L1) MFI on Day 1
log10(C_trachomatis_PorB antibody MFI)
y = c(0.692) + c(0.428) · x, r² = 0.34
R = 0.5, p = 3.4e−13
log10(Ct_PorB) MFI on Day 3
log10(Ct_PorB) MFI on Day 1
log10(KSHV_K81 antibody MFI)
y = c(0.48) + c(0.699) · x, r² = 0.488
R = 0.73, p < 2.2e−16
log10(KSHV_K81) MFI on Day 3
log10(KSHV_K81) MFI on Day 1
log10(HIV_gag antibody MFI)
y = c(0.331) + c(0.794) · x, r² = 0.742
R = 0.85, p < 2.2e−16
log10(HIV_gag) MFI on Day 3
log10(HIV_gag) MFI on Day 1
log10(HPV_16_E7 antibody MFI)
y = c(−0.121) + c(0.707) · x, r² = 0.455
R = 0.66, p < 2.2e−16
log10(HPV_16E7) MFI on Day 3
log10(HPV_16E7) MFI on Day 1
log10(HBV_HBc antibody MFI)
y = c(0.229) + c(0.589) · x, r² = 0.392
R = 0.59, p < 2.2e−16
log10(HBV_HBc) MFI on Day 3
log10(HBV_HBc) MFI on Day 1
log10(HPV_18_18L1 antibody MFI)
y = c(0.409) + c(0.715) · x, r² = 0.646
R = 0.75, p < 2.2e−16
log10(HPV_18L1) MFI on Day 3
log10(HPV_18L1) MFI on Day 1
log10(HCV_Core antibody MFI)
y = c(0.6) + c(0.547) · x, r² = 0.433
R = 0.63, p < 2.2e−16
log10(HCV_Core) MFI on Day 3
log10(HCV_Core) MFI on Day 1

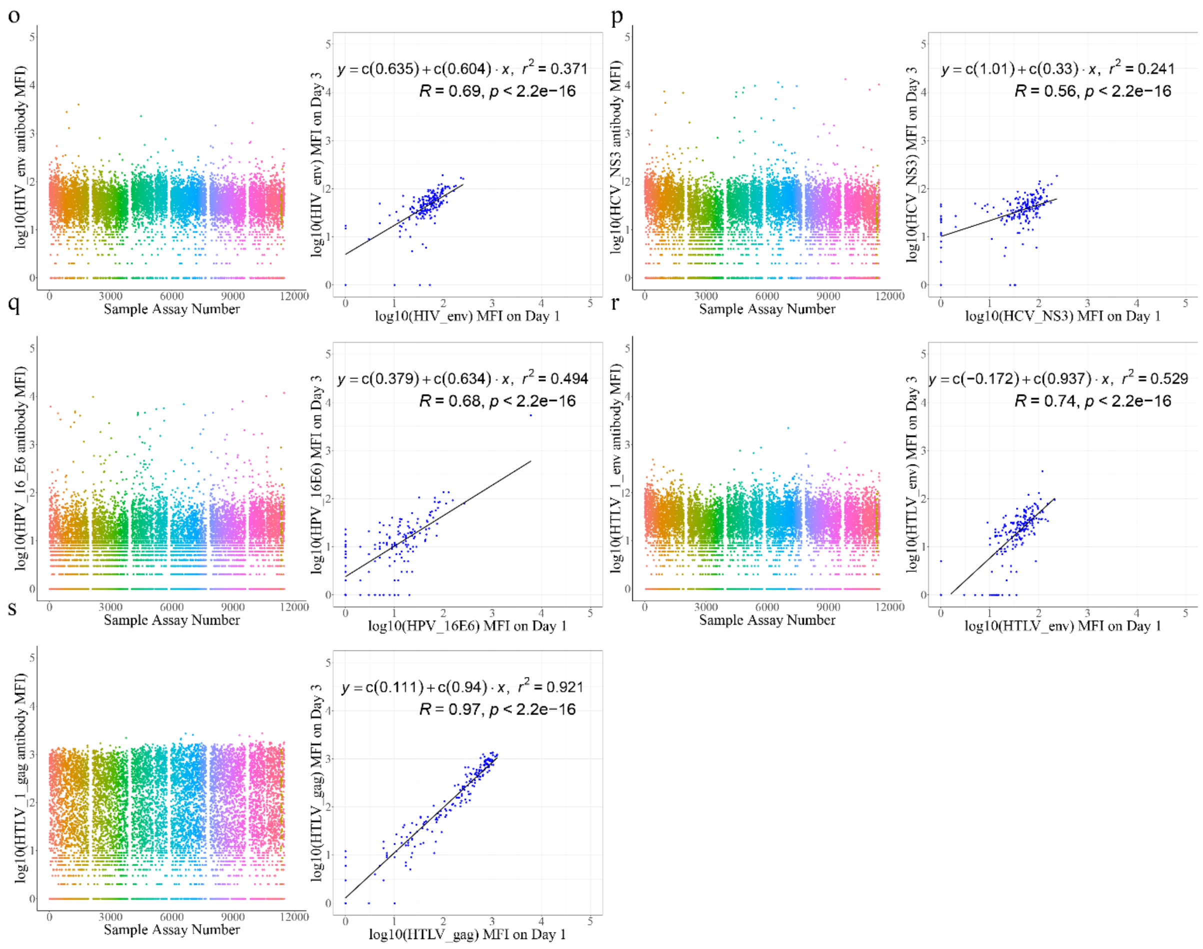

Supplementary Figure 4: Assessment of batch effects in the measurements of antibody responses to all 45 antigens in the UKB panel.

The log-transformed distribution of MFI values for all antigens tested in order of highest to lowest overall seroreactivity showing differences coloured by plate batch (n =110, left plot) and differences between absolute values on two separate days of assay using select bridging samples (right plot) with linear regression line and rho (R) and *P*-value from a two-sided t-test provided for A) EBV EA-D, B) BKV VP1, C) EBV VCAp18, D) HHV-7 U14, E) VZV gE/gI, F) EBV ZEBRA, G) EBV EBNA1, H) HHV-6 IE1A, I) HHV-6 IE1B, J) HSV-1 1gG, K) MCV VP1, L) CMV pp52, M) CMV pp150N, N) CMV pp28. O) JCV VP1, P) Hp GroEL, Q) Hp OMP, R) Ct TarpDF2, S) Hp CagA, T) Tg Sag1, U) HHV-6 p101k, V) Ct pGP3, W) Hp Urease, X) Ct TarpDF1, Y) Hp VacA, Z) Hp Catalase, a) Ct MompD, b) HSV-2 2mgGu, c) Tg p22, d) Ct MompA, e) HBV HBe, f) KSHV LANA, g) HPV-16 L1, h) Ct PorB, i) KSHV K8.1, j) HIV-1 Gag, k) HPV-16 E7, l) HBV HBc, m) HPV-18 L1, n) HCV Core, o) HIV-1 Env, p) HCV NS3, q) HPV-16 E6, r) HTLV-1 Env, s) HTLV-1 Gag. White spaces between batches represent duplicate samples (n=182) used for quality control at five points throughout the measurements.

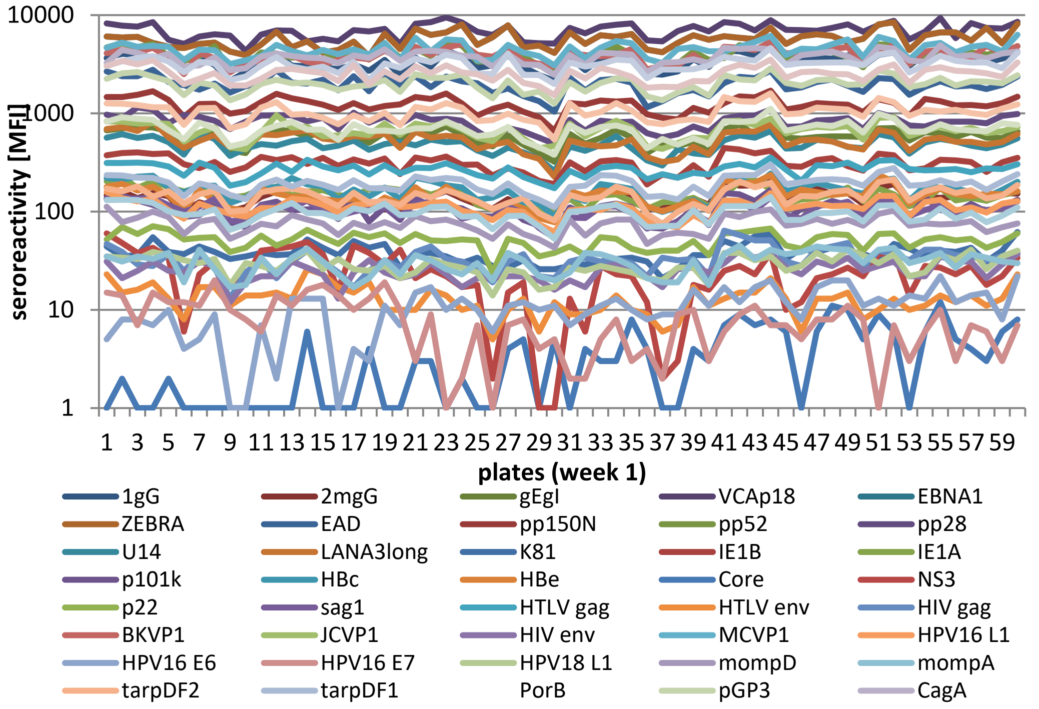

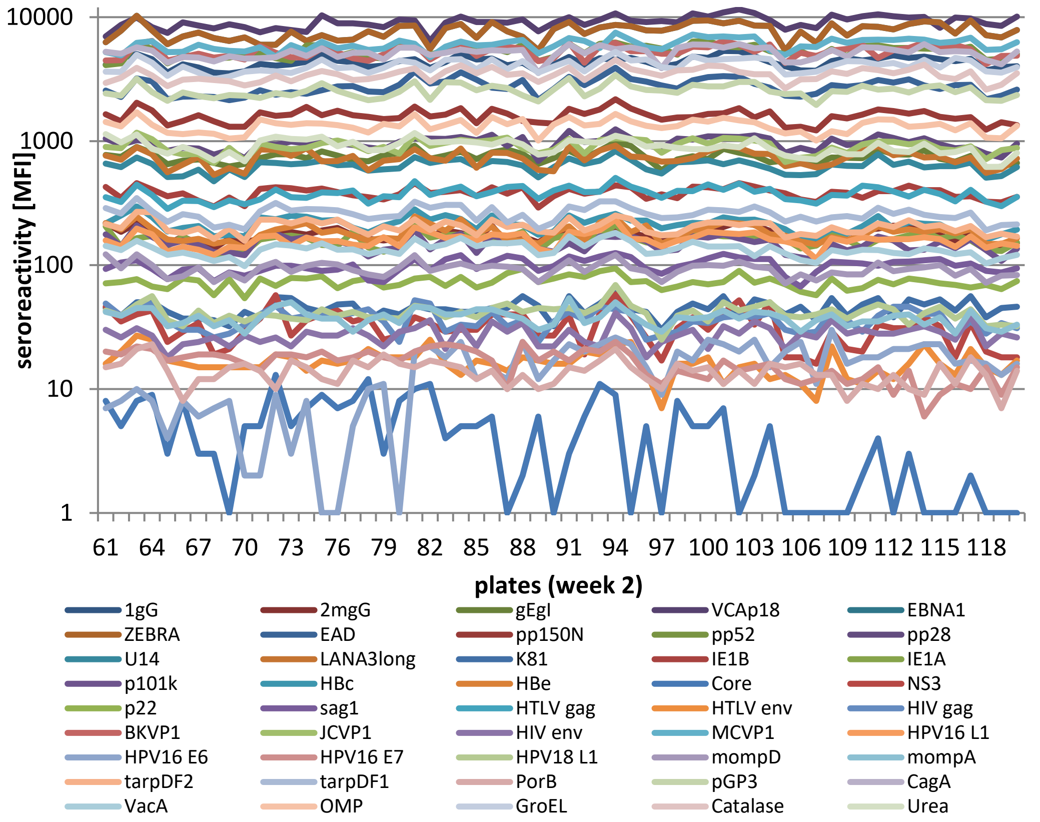

Supplementary Figure 5: Seroreactivity (MFI) of one control serum across all antigens and plates per assay week.

On each 96-well assay plate, three wells were used for control sera included on every plate to monitor plate handling. The figure exemplarily shows one of these control sera and its MFI reactivity against all 46 antigens (upper panel: week I comprising plates 1-60, lower panel: week II comprising plates 61 to 120).

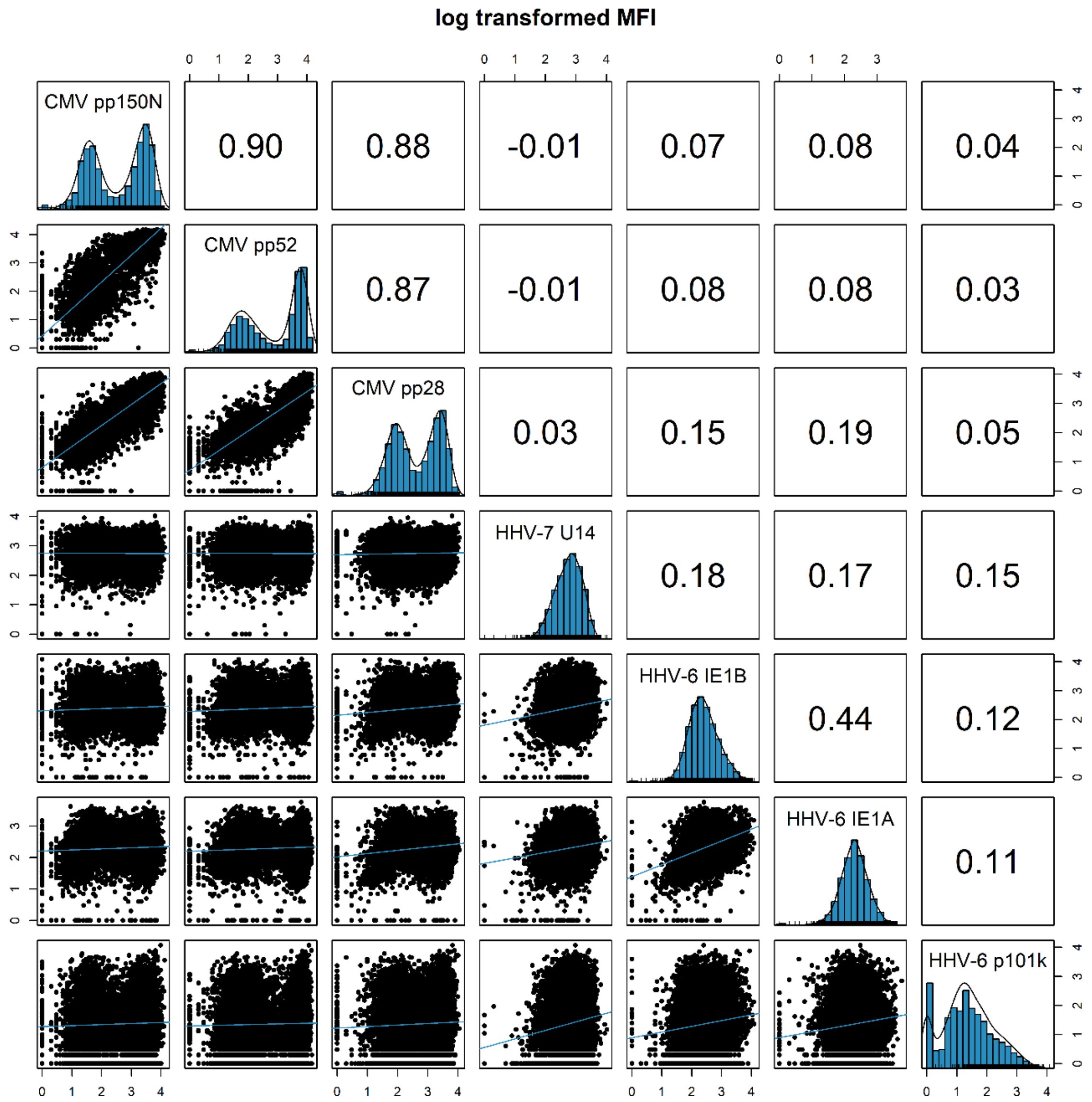

Supplementary Figure 6: Correlations between log-transformed antibody responses against beta herpesviridae antigens measured on the UKB Multiplex Serology platform.

The diagonal shows the histograms for the individual antibody responses. The lower panels represent the scatterplots of correlation between the two intersecting antigen responses with linear lines of best fit. The upper panel provides the measure of correlation (determined using Spearman rank) for the same two intersecting antibody responses.

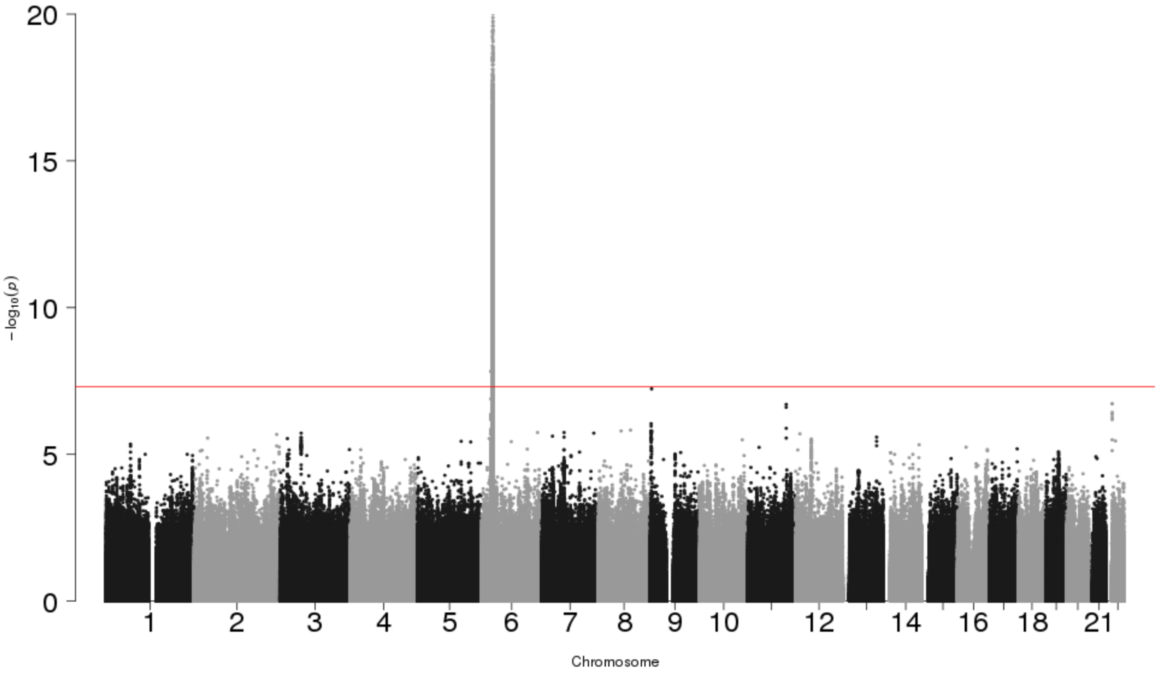

Supplementary Figure 7: Associations between imputed autosomal variants and $\log_{10}$ transformed quantitative responses against EBV VCAp18 antigen in 9,611 UKB participants.
The red line represents a threshold of genome-wide statistical significance ($P<5x10^{-8}$).

11. Supplementary Tables

Supplementary Table 1: Infectious agents considered and eventually taken forward for development on the Multiplex Serology platform.

Infectious Agent Considered	Putative Disease Links*	Taken forwards	Reason for exclusion	Antigens considered during validation stages	Final antigens incorporated into final Multiplex panel
HSV-1	Coronary heart disease, thyroid cancer, Alzheimer's disease	Yes	NA	1gD, 1gG	1gG
HSV-2	Prostate cancer, anogenital cancer	Yes	NA	2gD, 2mgGunique	2mgGunique
VZV	Leukaemia, lymphoma, skin cancer, breast cancer	Yes	NA	gE, gI, IE63	gE, gI
EBV	Lymphoproliferative disorders, gastric cancers, multiple sclerosis	Yes	NA	EBNA1, EA-D, ZEBRA, VCAp18	EBNA1, EA-D, ZEBRA, VCAp18
CMV	Glioblastoma, lymphoma, cervical, nasopharyngeal, colorectal cancers and cardiovascular diseases	Yes	NA	pp150N, pp52, pp28, pp65	pp150N, pp52, pp28
HHV-6A and 6B	Autoimmune and demyelinating diseases; lymphoma	Yes	NA	IE1A, IE1B, p101K, p100, MCP, U94A	IE1A, IE1B, p101K
HHV-7	Autoimmune and demyelinating diseases; lymphoma	Yes	NA	gB, U14	U14
KSHV	Lymphoproliferative cancers	Yes	NA	K8.1, LANA, ORF65	K8.1, LANA
HBV	Non-Hodgkin lymphoma and cholangiocarcinoma, type I diabetes	Yes	NA	HBc, HBe, HBs	HBc, HBe
HCV	Cholangiocarcinoma	Yes	NA	Core, NS3, NS4A, NS5A, NS5B	Core, NS3
HIV-1	Cardiovascular disease	Yes	NA	Gag, Env	Gag, Env
HTLV-1	Motor neuron disease, inflammatory arthropathy	Yes	NA	Gag, Env	Gag, Env
HPV-16	Cardiovascular disease, breast cancer	Yes	NA	L1, E6, E7	L1, E6, E7
HPV-18	Cardiovascular disease, breast cancer	Yes	NA	L1	L1
JCV	Prostate cancer	Yes	NA	VP1	VP1
BKV	bladder cancer	Yes	NA	VP1	VP1
MCV	Skin cancer	Yes	NA	VP1	VP1
T. gondii	Psychiatric conditions	Yes	NA	sag1, p22, bag1	sag1, p22
C. trachomatis	Prostate cancer	Yes	NA	mompA, mompD, tarpDF1, tarpDF2, PorB, Pgp3	mompA, mompD, tarpDF1, tarpDF2, PorB, Pgp3
H. pylori	Diverse gut cancers	Yes	NA	CagA-N, GroEL, Omp, VacA-C, Catalase, Urease	CagA-N, GroEL, Omp, VacA-C, Catalase, Urease
Human adenovirus D, serotype 36	Obesity	No	High risk of cross- reactivity with other adenoviridae	NA	NA
Influenza A and B	Cardiovascular disease	No	Limited ability to differentiate exposure *vs* vaccination; high risk of cross-reactivity with other serotypes	NA	NA

Coxsackie virus	Type I diabetes, sudden cardiac death	No	High risk of cross-reactivity with other enteroviruses	NA	NA
N. gonorrhoea	Cervical cancer	No	Difficulty identifying target antigens to guarantee assay specificity	NA	NA
S. aureus	Nil - Common bacterial infection	No	Difficulty identifying target antigens to guarantee assay specificity	NA	NA
B. burgdorferi	Lymphoma of skin	No	Difficulty identifying target antigens to guarantee assay specificity	NA	NA
Vaccine preventable diseases (*C. diphtheriae, C. tetani, B. pertussis,* measles, rubella, poliovirus, *S. pneumoniae, N. meningococcus*)	Nil	No	Lack of vaccination history in UKB and / or difficulty identifying target antigens to guarantee assay specificity	NA	NA

NA: not applicable.
* Examples represent a selection of diseases putatively linked to the infectious agent. Examples of confirmed associations are not included in this table.

Supplementary Table 2: Characteristics of infectious agent specific antigens used on the Multiplex Serology platform.

Infectious agent	Antigen	Likely Function	Gold standard assay	Reference
HSV-1	1gG	membrane glycoprotein	Enzygnost anti-HSV IgG (Siemens Healthcare Diagnostics)	Brenner et al [7]
HSV-2	2mgGunique	membrane glycoprotein	LIAISON HSV-2 Type Specific IgG (DiaSorin)	Brenner et al [7]
VZV	gE/gI*	envelope glycoproteins	Time-resolved fluorescence immunoassay (TRFIA)	Brenner et al [7]
EBV	VCAp18	capsid protein	Enzygnost anti-EBV IgG (Siemens Healthcare Diagnostics GmbH)	Brenner et al [7]
	EBNA1	replication, latent viral infection		
	ZEBRA	replication activator		
	EA-D	replication (polymerase accessory subunit)		
CMV	pp150 (N-terminus)	tegument protein	Enzygnost anti-CMV IgG (Siemens Healthcare Diagnostics); ELISA: Stanley Neurovirology Laboratory (John Hopkins University)	Brenner et al [7]
	pp52	DNA binding protein		
	pp28	capsid protein		
HHV-6**	IE1B	potential transactivator	NA	Bassig et al[32], Engdahl et al[33], Freuer et al[34]
	IE1A	potential transactivator		
	p101k	potential tegument protein		
HHV-7**	U14	potential tegument protein	NA	Validation ongoing
KSHV**	LANA	replication and long-term persistence	NA	Validation ongoing
	K8.1	structural glycoprotein		
HBV	HBc	core antigen	ARCHITECT anti-HBc (Abbott Laboratories, Abbott Park, USA); ARCHITECT anti-HBc II (Abbott Laboratories)	Brenner et al [8]
	HBe	soluble nucleocapsid associated antigen		
HCV	Core	structural antigen	ARCHITECT Anti-HCV (Abbott Laboratories), confirmatory ELISA Murex anti-HCV (Diasorin, Saluggia, Italy); AxSYM HCV version 3.0 microparticle enzyme immune assay (MEIA; Abbott Diagnostics); Western blot (INNO-LIA HCV SNS3; Innogenetics)	Dondog et al [35], Brenner et al [8]
	NS3	protease and RNA helicase activity		
T. gondii	p22	surface protein	Sabin-Feldman dye test	Brenner et al [8]
	sag1	surface protein		
HTLV-1	Gag	structural antigen	ARCHITECT rHTLV-I/II (Abbott Laboratories), confirmation and typing by Genelabs Diagnositics HTLV 2.4 assay (Genelabs, Redwood City, USA)	Brenner et al [8]
	Env	structural antigen		
HIV-1	Gag	structural antigen	ARCHITECT HIV Ag/Ab Combo assay (Abbott); INNO-LIA HIV I/II Score assay (Innogenetics)	Kranz et al [9]

	Env	structural antigen		
BKV	VP1	major capsid protein	NA***	Kjaerheim et al [21], Gossai et al [20], Robles et al [22]
JCV	VP1	major capsid protein	NA***	Kjaerheim et al [21], Gossai et al [20], Robles et al [22]
MCV	VP1	major capsid protein	NA***	Gossai et al [20], Robles et al [22]
HPV-16	L1	major capsid protein	NA***	Petter et al[36], Sehr et al [3, 4]
	E6	oncogen	NA***	
	E7	oncogen	NA***	
HPV18	L1	major capsid protein	NA***	Petter et al[36], Sehr et al [3, 4]
C. trachomatis	mompD	structural antigen	Medac Chlamydia trachomatis-IgG-pElisa (Ct-ELISA; Medac, Wedel, Germany)	Hulstein et al [37], Trabert et al [10], Wills et al[38]
	mompA	structural antigen		
	TarpDF1	regulation of actin recruitment		
	TarpDF2			
	PorB	porin		
	pGP3	virulence factor	Public Health England pGP3 reference assay	
H. pylori	CagA (N-terminus)	pathogenesis	Ridascreen Helicobacter IgA, IgG (R-Biopharm, Darmstadt, Germany); P120 CagA-ELISA (Ravo Diagnostika, Freiburg, Germany); Helicobacter-ELISA (Dr. Fenning BioMed, Kirchzarten, Germany); Helicobacter pylori ViraBlot (Viramed Biotech GmbH, Planegg, Germany)	Michel et al [39]
	VacA (C-terminus)	pathogenesis		
	OMP	cell envelope		
	GroEL	chaperonin		
	Catalase	detoxification		
	UreA	urease alpha subunit		
-	GST	used for background subtraction	NA	Sehr et al [4]

*During the validation stages optimal sensitivity and specificity metrics were achieved for VZV when simultaneously loading gE and gI antigens to the same bead sets. These were the only antigens loaded in such a way in the Multiplex Serology panel.
**For HHV 6-8 antigens, the following sequences were expressed: IE1A: 382-683 aa (Uniprot: M9TM52); IE1B: 400-775 aa (Uniprot: Q77PU6); p101k: 282-395 aa (Uniprot: Q69535); U14: 1-648 aa (Uniprot: Q69495); LANA: 863-1129 aa (NCBI: NC_009333); K8.1: 1-228 aa (NCBI: NC_009333).
*** These assays have been extensively used in previously published seroepidemiological studies.
NA, not applicable.

Supplementary Table 3: Intra-class correlation coefficients (ICC) comparing Multiplex Serology assays on non-magnetic and magnetic beads.

Infectious agent	ICC* (95%CI)
HSV-1	0.95 (0.93-0.96)
HSV-2	0.96 (0.94-0.97)
VZV	0.87 (0.83-0.90)
EBV	0.89 (0.86-0.92)
CMV	0.99 (0.99-0.99)
HIV-1	0.83 (0.79-0.87)
HTLV-1	0.92 (0.90-0.94)
HBV	1.0 (1.0-1.0)
HCV	0.96 (0.94-0.97)
T. gondii	0.48 (0.36-0.59)

*: ICCs were interpreted as follows: 0.01<ICC<0.49: poor reliability, 0.50<ICC<0.74: moderate reliability, 0.75<ICC<0.89: good reliability, 0.90<ICC<1.00: excellent reliability.

Supplementary Table 4: Correlation between tested bridging panel samples (n=182) on multiple assay days for each antigen.
Spearman Rho correlation coefficients are presented for each set of days comparison for each antigen. The Day1 vs Day3 Comparison has plots shown in Supplementary Figure 4.

Antigen	Day1 vs Day2	Day1 vs Day3	Day1 vs Day4	Day4 vs Day5	Day5 vs Day6
HSV-1 1gG	0.9	0.92	0.93	0.94	0.96
HSV-2 2mgGu	0.92	0.93	0.86	0.88	0.91
VZV gE/gI	0.94	0.95	0.94	0.97	0.98
EBV VCAp18	0.5	0.59	0.64	0.8	0.85
EBV ZEBRA	0.85	0.86	0.85	0.89	0.92
EBV EBNA1	0.91	0.92	0.92	0.93	0.96
EBV EA-D	0.95	0.96	0.95	0.97	0.98
CMV pp150N	0.96	0.96	0.95	0.96	0.96
CMV pp52	0.89	0.92	0.94	0.94	0.96
CMV pp28	0.96	0.96	0.96	0.95	0.94
HHV-7 U14	0.91	0.92	0.92	0.94	0.97
KSHV LANA	0.81	0.81	0.74	0.8	0.81
KSHV K8.1	0.69	0.73	0.72	0.82	0.83
HHV6_IE1B	0.96	0.95	0.94	0.92	0.91
HHV-6 IE1A	0.89	0.87	0.83	0.77	0.78
HHV-6 p101k	0.92	0.92	0.84	0.92	0.92
HBV HBc	0.51	0.59	0.44	0.62	0.7
HBV HBe	0.74	0.74	0.8	0.8	0.81
HCV Core	0.63	0.63	0.61	0.68	0.43
HCV NS3	0.55	0.56	0.56	0.61	0.67
Tg p22	0.87	0.87	0.84	0.86	0.85
Tg Sag1	0.87	0.89	0.63	0.82	0.89
HTLV-1 Gag	0.95	0.97	0.96	0.98	0.97
HTLV-1 Env	0.66	0.74	0.73	0.71	0.77
HIV-1 Gag	0.85	0.85	0.88	0.87	0.84
HIV-1 Env	0.69	0.69	0.67	0.75	0.79
JCV VP1	0.87	0.89	0.88	0.93	0.95
BKV VP1	0.97	0.97	0.95	0.98	0.98
MCV VP1	0.95	0.96	0.96	0.97	0.98
HPV-16 L1	0.79	0.84	0.82	0.79	0.81
HPV-16 E6	0.66	0.68	0.58	0.55	0.56
HPV-16 E7	0.72	0.66	0.79	0.82	0.82
HPV-18 L1	0.72	0.75	0.78	0.8	0.83
Ct MompD	0.79	0.8	0.76	0.84	0.88
Ct MompA	0.71	0.71	0.74	0.77	0.79
Ct TarpDF2	0.91	0.9	0.87	0.91	0.92
Ct TarpDF1	0.86	0.87	0.84	0.9	0.9
Ct PorB	0.52	0.5	0.44	0.67	0.77
Ct pGP3	0.78	0.78	0.79	0.79	0.86
Hp CagA	0.95	0.96	0.5	0.97	0.98
Hp VacA	0.89	0.88	0.82	0.82	0.87
Hp OMP	0.94	0.95	0.94	0.93	0.92
Hp GroEL	0.92	0.93	0.85	0.83	0.84
Hp Catalase	0.91	0.89	0.84	0.87	0.89
Hp Urease	0.94	0.95	0.91	0.92	0.92

Supplementary Table 5: Coefficients of variation (CV) for blind spiked duplicates (n=107) from samples from UKB.

Antigen	GMT Duplicate 1 (95%CI)*	GMT Duplicate 2 (95%CI) *	CV in all samples	CV in seropositives only**
**HSV-1 1gG**	891 (859-925)	723 (695-752)	9.7	4.1
**HSV-2 2mgGu**	41 (40-42)	34 (33-35)	16.3	8.8
**VZV gE/gI**	581 (568-593)	472 (460-484)	14.4	5.2
**EBV VCAp18**	4968 (4828-5113)	4270 (4139-4406)	12.8	4.4
**EBV EBNA1**	2238 (2158-2321)	1817 (1745-1892)	11.3	3.7
**EBV ZEBRA**	1211 (1178-1245)	951 (921-983)	15.7	7.3
**EBV EA-D**	1200 (1163-1238)	965 (930-1002)	16.6	3.5
**CMV pp150N**	324 (312-337)	257 (247-268)	14.5	3.7
**CMV pp52**	583 (558-608)	446 (426-468)	17.3	6.5
**CMV pp28**	388 (375-401)	312 (301-323)	18.8	3.2
**HHV-7 U14**	567 (558-577)	459 (448-469)	13.3	3.4
**KSHV LANA**	14 (14-15)	12 (11-12)	18.0	2.6
**KSHV K8.1**	50 (49-51)	44 (44-45)	14.8	10.4
**HHV-6 IE1B**	291 (284-297)	203 (198-209)	20.4	7.3
**HHV-6 IE1A**	236 (233-240)	166 (162-170)	26.2	6.0
**HHV-6 p101k**	33 (32-35)	27 (27-28)	24.8	5.8
**HBV HBc**	6 (6-6)	5 (5-5)	10.3	0.9
**HBV HBe**	13 (13-13)	11 (11-12)	18.3	2.2
**HCV Core**	5 (5-6)	5 (5-5)	22.7	NA
**HCV NS3**	25 (24-25)	22 (21-22)	25.5	NA
***Tg* p22**	37 (36-38)	30 (29-31)	20.3	2.0
***Tg* sag1**	85 (83-86)	70 (69-72)	16.6	1.4
**HTLV-1 Gag**	60 (58-62)	51 (49-52)	16.7	NA
**HTLV-1 Env**	24 (23-24)	19 (18-19)	17.0	NA
**HIV-1 Gag**	64 (63-65)	49 (48-50)	16.5	0.3
**HIV-1 Env**	33 (32-33)	26 (26-27)	12.9	NA
**BKV VP1**	2519 (2470-2569)	2043 (1994-2094)	13.8	7.6
**JCV VP1**	373 (364-382)	299 (291-307)	20.8	3.1
**MCV VP1**	733 (709-758)	585 (565-606)	13.7	2.5
**HPV-16 L1**	39 (38-40)	31 (30-31)	15.2	7.2
**HPV-16 E6**	12 (11-12)	9 (8-9)	14.1	NA
**HPV-16 E7**	18 (18-19)	15 (15-15)	18.7	2.2
**HPV-18 L1**	37 (36-38)	30 (30-31)	17.4	7.2
***Ct* MompD**	25 (24-26)	17 (16-17)	25.0	2.8
***Ct* MompA**	25 (24-26)	18 (17-18)	18.7	2.8
***Ct* TarpDF2**	37 (36-39)	30 (29-31)	24.0	3.3
***Ct* TarpDF1**	21 (20-22)	17 (17-18)	15.1	2.5
***Ct* PorB**	13 (13-13)	11 (11-12)	19.1	0.2
***Ct* pGP3**	23 (22-24)	21 (20-21)	26.5	12.0
***Hp* CagA**	139 (133-145)	134 (128-140)	7.8	3.0
***Hp* VacA**	38 (37-39)	28 (28-29)	18.7	3.7
***Hp* OMP**	85 (82-88)	64 (61-66)	22.5	3.3
***Hp* GroEL**	36 (35-38)	26 (25-28)	35.4	4.0
***Hp* Catalase**	61 (59-63)	49 (47-50)	18.4	3.5
***Hp* Urea**	47 (46-49)	39 (38-41)	16.3	4.4

*GMT: geometric mean titre for 107 individuals with blind spiked duplicate samples available from the same encounter.
**coefficient of variation calculated only when both duplicates were defined as seropositive. For some antigens that were observed at low frequencies, these calculations were not possible (NA).

Supplementary Table 6: Seroconversion and reversion rates estimated from 277 individuals with samples available at both baseline and repeat assessment encounters.

Infectious agent	Seroconversion (%)	Seroreversion (%)	Mean of log_{10} distribution of 277 repeat measures*	Mean (sd) of log_{10} distribution of only sero-converters or reverters†	Proportion of seroconverters or reverters within 1sd†
HSV-1	14 (5.0)	11 (4.0)	2.9	2.1 (0.5)	80.0
HSV-2	10 (3.6)	6 (2.2)	1.6	2.2 (0.5)	84.4
VZV	12 (4.3)	5 (1.8)	2.7	2.1 (0.4)	73.5
EBV	1 (0.4)	1 (0.4)	-	-	-
CMV	14 (5.0)	6 (2.2)	-	-	-
HHV-6A or 6B	22 (7.9)	19 (6.9)	-	-	-
HHV-7	4 (1.4)	6 (2.2)	-	-	-
KSHV	7 (2.5)	4 (1.4)	-	-	-
HBV	2 (0.7)	0	-	-	-
HCV	0	1 (0.4)	-	-	-
HIV-1	0	0	-	-	-
HTLV-1	0	1 (0.4)	-	-	-
HPV-16	3 (1.1)	5 (1.8)	1.5	2.2 (0.2)	75.0
HPV-18	4 (1.4)	1 (0.4)	1.4	2.1 (0.3)	80.0
JCV	19 (6.9)	23 (8.3)	2.6	2.4 (0.2)	75.0
BKV	6 (2.2)	6 (2.2)	3.4	2.5 (0.2)	83.3
MCV	21 (7.6)	12 (4.3)	2.9	2.3 (0.6)	86.3
T. gondii	29 (10.5)	26 (9.4)	-	-	-
C. trachomatis	1 (0.4)	3 (1.1)	-	-	-
H. pylori‡	19 (6.9)	11 (4.0)	-	-	-

*: No standard deviations (sd) presented as the 277 repeat samples were not always normally distributed.

†: Standard deviations (sd) presented as seroconverters or reverters alone were always approximately normally distributed. Distributions only compared for those infectious agents where seroprevalence determined using a single antigen.

‡: Estimates calculated from 50% of samples.

Supplementary Table 7: Unadjusted seroprevalence estimates and 95% confidence intervals for all antigens tested using the Multiplex Serology platform in the 9,695 individuals from UKB overall, and stratified by sex. Estimates highlighted in bold have non-overlapping 95% confidence interval (CI) ranges between sexes.

Antigen	Sero-reactivity (%)	95% CI	Sero-reactivity males (%)	95% CI males	Sero-reactivity females (%)	95% CI females
HSV-1 1gG	69.8	68.9-70.7	68.7	67.3-70.1	70.7	69.5-72.0
HSV-2 2mgGu	16.2	15.5-16.9	15.3	14.2-16.4	16.9	15.9-17.9
VZV gE/gI	92.5	92.0-93.0	**94.0**	**93.2-94.7**	**91.4**	**90.6-92.1**
EBV VCAp18	94.9	94.5-95.4	**93.7**	**93.0-94.4**	**96.0**	**95.4-96.4**
EBV ZEBRA	91.1	90.6-91.7	**88.2**	**87.3-89.2**	**93.4**	**92.7-94.1**
EBV EBNA1	88.8	88.2-89.4	87.8	86.9-88.8	89.6	88.8-90.4
EBV EA-D	96.1	95.4-96.8	**83.1**	**81.9-84.2**	**88.6**	**87.7-89.4**
CMV pp150N	59.4	58.4-60.3	**57.3**	**55.9-58.8**	**61.0**	**59.7-62.3**
CMV pp52	64.9	64.0-65.9	64.9	63.5-66.4	64.9	63.6-66.2
CMV pp28	58.6	57.6-59.5	57.8	56.4-59.3	59.1	57.8-60.4
HHV-6 IE1B	79.6	78.7-80.3	80.2	79.0-81.4	79.0	77.9-80.1
HHV-6 IE1A	77.8	76.9-78.6	**79.7**	**78.5-81.0**	**76.2**	**75.1-77.3**
HHV-6 p101k	22.3	21.5-23.2	**19.1**	**17.9-20.3**	**24.9**	**23.7-26.0**
HHV-7 U14	94.7	94.3-95.2	**92.9**	**92.1-93.7**	**96.2**	**95.7-96.7**
KSHV LANA	5.8	5.3-6.3	6.4	5.7-7.2	5.3	4.7-5.9
KSHV K8.1	3.2	2.8-3.5	3.5	3.0-4.1	2.9	2.5-3.4
HBV HBc	3.0	2.7-3.3	**3.9**	**3.3-4.4**	**2.3**	**1.9-2.7**
HBV HBe	6.2	5.7-6.7	6.8	6.1-7.6	5.7	5.1-6.4
HCV Core	2.0	1.8-2.3	2.5	2.0-3.0	1.7	1.3-2.0
HCV NS3	1.4	1.1-1.6	**1.8**	**1.4-2.2**	**1.0**	**0.7-1.3**
HIV-1 Gag	3.2	2.8-3.5	3.5	3.0-4.1	2.9	2.5-3.4
HIV-1 Env	1.5	1.2-1.7	1.5	1.1-1.9	1.5	1.2-1.8
HTLV-1 Gag	0.7	0.5-0.9	**1.2**	**0.9-1.5**	**0.3**	**0.2-0.5**
HTLV-1 Env	0.9	0.7-1.1	0.9	0.6-1.1	1.0	0.7-1.3
HPV-16 L1	4.4	4.0-4.8	**2.7**	**2.2-3.2**	**5.7**	**5.1-6.4**
HPV-16 E6	1.0	0.8-1.2	1.0	0.7-1.3	0.9	0.7-1.2
HPV-16 E7	3.2	2.8-3.5	3.7	3.1-4.2	2.8	2.3-3.2
HPV-18 L1	2.7	2.4-3.1	**1.9**	**1.5-2.4**	**3.4**	**2.9-3.8**
JCV VP1	57.5	56.5-58.5	**60.9**	**59.4-62.4**	**54.8**	**53.5-56.2**
BKV VP1	95.4	95.0-95.8	**96.1**	**95.5-96.6**	**94.8**	**94.2-95.4**
MCV VP1	66.7	65.8-67.7	67.7	66.3-69.1	66.0	64.7-67.2
Tg p22	15.0	14.3-15.7	**16.5**	**15.3-17.6**	**13.9**	**12.9-14.8**
Tg Sag1	22.8	22.0-23.7	23.2	21.9-24.4	22.6	21.5-23.7
Ct MompD	16.3	15.6-17.0	**13.7**	**12.6-14.7**	**18.3**	**17.3-19.4**
Ct MompA	11.6	10.9-12.2	**9.7**	**8.8-10.6**	**13.1**	**12.2-13.9**
Ct TarpDF2	23.8	22.9-24.6	**21.6**	**20.4-22.8**	**25.5**	**24.4-26.7**
Ct TarpDF1	19.6	18.8-20.3	**16.5**	**15.4-17.6**	**22.0**	**20.9-23.1**
Ct PorB	3.4	3.0-3.7	**2.4**	**2.0-2.9**	**4.1**	**3.6-4.6**
Ct pGP3	21.4	20.6-22.2	**15.9**	**14.8-17.0**	**25.7**	**24.5-26.9**
Hp CagA*	23.2	22.1-24.4	22.8	21.0-24.6	23.5	22.0-25.1
Hp VacA*	19.2	18.4-20.0	**21.1**	**19.9-22.3**	**17.6**	**16.6-18.7**
Hp OMP*	31.1	30.2-32.1	**33.4**	**32.0-34.9**	**29.3**	**28.1-30.5**
Hp GroEL*	31.7	30.8-32.7	**34.2**	**32.8-35.6**	**29.8**	**28.6-31.0**
Hp Catalase*	17.8	17.1-18.6	**20.1**	**18.9-21.3**	**16.0**	**15.1-17.0**
Hp Urease*	21.2	20.4-22.0	**24.4**	**23.1-25.7**	**18.7**	**17.7-19.7**

*: Estimates calculated from 50% of samples.

Supplementary Table 8: Crude seroprevalence estimates and adjusted odds ratio of antibody seroprevalence in the UKB subset.

Agents presented include those with non-overlapping 95% confidence intervals (CI) of crude estimates by sex from Table 2.

Agent	Sex*	Number Seropositive	Crude Seroprevalence (95%CI)	Adjusted OR (95%CI) †	Adjusted p-value†
**VZV**					
	Male	4013	94.0 (93.2-94.7)		
	Female	4957	91.4 (90.6-92.1)	0.62 (0.51-0.74)	$1.5x10^{-7}$
**EBV**					
	Male	3991	93.4 (92.7-94.2)		
	Female	5191	95.7 (95.2-96.2)	2.06 (1.67-2.53)	$9.2x10^{-12}$
**HHV-7**					
	Male	3967	92.9 (92.1-93.7)		
	Female	5218	96.2 (95.7-96.7)	1.97 (1.61-2.42)	$6.7x10^{-11}$
**HBV**					
	Male	142	3.3 (2.9-3.9)		
	Female	97	1.8 (1.4-2.1)	0.54 (0.38-0.75)	$3.2x10^{-4}$
**HTLV-1**					
	Male	86	2.0 (1.6-2.4)		
	Female	66	1.0 (0.9-1.5)	0.60 (0.42-0.85)	$4.7x10^{-3}$
**HPV-16**					
	Male	116	2.7 (2.2-3.2)		
	Female	311	5.7 (5.1-6.4)	2.84 (2.20-3.83)	$1.5x10^{-15}$
**HPV-18**					
	Male	83	1.9 (1.5-2.4)		
	Female	182	3.4 (2.9-3.8)	2.21 (1.62-3.01)	$5.5x10^{-7}$
**JCV**					
	Male	2601	60.9 (59.4-62.4)		
	Female	2974	54.8 (53.5-56.2)	0.77 (0.71-0.85)	$5.8x10^{-8}$
**BKV**					
	Male	4103	96.1 (95.5-96.7)		
	Female	5144	94.8 (94.2-95.4)	0.76 (0.60-0.96)	$5.1x10^{-7}$
***Hp*‡**					
	Male	792	38.2 (36.1-40.3)		
	Female	927	33.1 (31.4-34.9)	0.77 (0.67-0.88)	$1.6x10^{-4}$
Ct					
	Male	679	15.9 (14.8-17.0)		
	Female	1394	25.7 (24.5-26.9)	2.73 (2.39-3.1)	$5.34x10^{-51}$

*: male sex (n=4271; 2071 for *Hp*) used as reference for female (n=5424; 2800 for *Hp*).

†: odds ratios (OR) and *P*-values calculated using multivariable logistic regression adjusted for age interval, TDI, self-reported ethnicity, LSP and sameSI.

‡: only individuals tested in the first week of analysis were included.

Supplementary Table 9: Unadjusted seroprevalence estimates and adjusted odds ratios of infectious agent seroprevalence in the UKB sample clustered by age intervals and stratified by sex.
Agents reported represent those with significant (*P*<0.01) evidence of differences based on crude estimates and with sufficient numbers of individuals available for models to converge.

**Agent**	**Age Category**†	**Number positive males**	**Seroprevalence males (95%CI)**	**Adjusted OR males (95%CI)**	***P*-value* males**	**Number positive females**	**Seroprevalence females (95% CI)**	**Adjusted OR females (95%CI)**	***P*-value* females**
**HSV-1**									
	40-50	696	64.0 (61.1-66.8)			986	67.8 (65.4-70.2)		
	50-60	974	67.1 (64.7-69.5)	**1.22 (1.01-1.47)**	**0.03**	1338	69.0 (66.9-71.1)	1.13 (0.96-1.33)	NS
	60-70	1263	72.9 (70.8-75.0)	**1.81 (1.49-2.18)**	**$1.0x10^{-9}$**	1513	74.5 (72.6-76.4)	**1.69 (1.42-2.00)**	**$3.2x10^{-9}$**
**HSV-2**									
	40-50	177	16.3 (14.1-18.5)			289	19.9 (17.8-21.9)		
	50-60	215	14.8 (13.0-16.6)	1.02 (0.79-1.31)	NS	330	17.0 (15.3-18.7)	1.08 (0.88-1.32)	NS
	60-70	262	15.1 (13.4-16.8)	1.16 (0.90-1.49)	NS	297	14.6 (13.1-16.2)	1.15 (0.93-1.43)	NS
**VZV**									
	40-50	998	91.7 (90.1-93.3)			1330	91.4 (90.0-92.8)		
	50-60	1363	93.9 (92.7-95.2)	1.28 (0.90-1.82)	NS	1756	90.6 (89.3-91.9)	0.95 (0.73-1.23)	NS
	60-70	1652	95.4 (94.4-96.4)	**1.78 (1.22-2.58)**	**$2.6x10^{-3}$**	1871	92.2 (91.0-93.3)	1.08 (0.82-1.43)	NS
**CMV**									
	40-50	518	47.6 (44.6-50.6)			716	49.2 (46.6-51.8)		
	50-60	832	57.3 (54.8-60.0)	**1.59 (1.33-1.91)**	**$4.0x10^{-7}$**	1146	59.1 (56.9-61.3)	**1.66 (1.42-1.94)**	**$9.5x10^{-11}$**
	60-70	1076	62.1 (59.8-64.4)	**1.95 (1.63-2.34)**	**$2.8x10^{-13}$**	1357	66.8 (64.8-68.9)	**2.52 (2.14-2.96)**	**$1.3x10^{-28}$**
**HPV-16**									
	40-50	35	3.8 (2.6-4.9)			126	8.7 (7.2-10.1)		
	50-60	40	2.4 (1.6-3.2)	0.81 (0.47-1.38)	NS	121	6.2 (5.2-7.3)	0.81 (0.60-1.08)	NS
	60-70	23	2.3 (1.6-3.0)	0.82 (0.48-1.42)	NS	64	3.2 (2.4-3.9)	**0.50 (0.35-0.72)**	**$2.0x10^{-4}$**
**HPV-18**									
	40-50	23	2.1 (1.3-3.0)			74	5.1 (4.0-6.2)		
	50-60	29	2.0 (1.3-2.7)	1.10 (0.58-2.08)	NS	62	3.2 (2.4-4.0)	0.84 (0.57-1.24)	NS
	60-70	31	1.8 (1.2-2.4)	0.86 (0.43-1.68)	NS	46	2.3 (1.6-2.9)	0.71 (0.45-1.10)	NS
**JCV**									
	40-50	672	61.8 (58.9-64.7)			857	58.9 (56.4-61.4)		
	50-60	892	61.5 (59.0-64.0)	1.07 (0.90-1.29)	NS	1043	53.8 (51.6-56.0)	**0.83 (0.71-0.97)**	**0.02**
	60-70	1732	60.0 (57.6-62.2)	0.97 (0.82-1.16)	NS	1074	52.9 (50.7-55.1)	**0.78 (0.66-0.91)**	**$1.8x10^{-3}$**
**BKV**									
	40-50	1060	97.4 (96.5-98.4)			1411	97.0 (96.1-97.9)		
	50-60	1396	96.2 (95.2-97.2)	0.75 (0.45-1.26)	NS	1846	95.2 (94.3-96.2)	0.68 (0.44-1.02)	
	60-70	1647	95.1 (94.1-96.1)	**0.53 (0.32-0.87)**	**0.01**	1887	93.0 (91.8-94.1)	**0.40 (0.27-0.59)**	**$8.5x10^{-6}$**
**MCV**									
	40-50†	745	68.5 (65.7-71.2)			997	68.5 (66.1-70.9)		
	50-60	991	68.3 (65.9-70.7)	1.09 (0.90-1.31)	NS	1302	67.1 (65.1-69.2)	0.97 (0.83-1.14)	NS
	60-70	1154	66.6 (64.4-68.8)	1.02 (0.85-1.23)	NS	1279	63.0 (60.9-65.1)	0.87 (0.74-1.02)	NS
Tg									
	40-50	259	23.8 (21.3-26.3)			350	24.1 (21.6-26.3)		
	50-60	421	29.0 (26.7-31.3)	**1.42 (1.15-1.74)**	**$7.5x10^{-4}$**	493	25.4 (23.5-27.4)	**1.22 (1.02-1.45)**	**0.03**
	60-70	559	32.3 (30.1-34.5)	**1.62 (1.32-1.98)**	**$2.9x10^{-6}$**	631	31.1 (29.1-33.1)	**1.59 (1.33-1.90)**	**$4.0x10^{-6}$**
Hp‡									
	40-50	185	35.1 (31.0-39.2)			217	28.4 (25.2-31.6)		
	50-60	255	36.2 (32.7-39.8)	1.12 (0.86-1.47)	NS	349	34.3 (31.4-37.2)	**1.54 (1.22-1.95)**	**$3.3x10^{-4}$**
	60-70	352	41.9 (38.6-45.2)	**1.50 (1.15-1.96)**	**$2.7x10^{-3}$**	361	35.4 (32.5-38.3)	**1.53 (1.20-1.96)**	**$6.9x10^{-4}$**
Ct									
	40-50	216	19.9 (17.5-22.2)			458	31.5 (29.1-33.9)		
	50-60	239	16.5 (14.6-18.4)	0.97 (0.76-1.26)	NS	550	28.4 (26.4-30.4)	**1.29 (1.07-1.54)**	**$6.2x10^{-3}$**
	60-70	224	12.9 (11.4-14.5)	1.03 (0.80-1.33)	NS	386	19.0 (17.3-20.7)	0.99 (0.81-1.21)	NS

*: Odds ratio (OR) and *P*-values calculated using multivariable logistic regression reported after adjustment for TDI, self-reported ethnicity, LSP and sameSI. Significant associations are highlighted in bold. NS: not significant.
†: Age category 40-50 used as reference in models (n_{males}=1088, $n_{females}$=1455) compared against 50-60 (n_{males}=1451, $n_{females}$=1939) and 60-70 (n_{males}=1732, $n_{females}$=2030).
‡: Samples only available for week 1; n_{males} by category 527, 704, 840; $n_{females}$ 763, 1017, 1020.

Supplementary Table 10: Unadjusted seroprevalence estimates and adjusted odds ratio of seroprevalence for multiple infectious agents in the UKB subset clustered by self-reported ethnicity and stratified by sex. Agents reported represent those with significant evidence (*P*<0.01) of association based on crude estimates.

Agent	Ethnicity	Number positive males	Seroprevalence males (95%CI)	Adjusted OR males (95%CI)	*P*-value*	Number positive females	Seroprevalence females (95% CI)	Adjusted OR females (95%CI)	*P*-value* females
**HSV-1**									
	White	2717	67.8 (66.3-69.2)			3602	70.2 (68.9-71.4)		
	Asian	99	81.1 (74.2-88.0)	**2.06 (1.20-3.54)**	**8.6x10^{-3}**	85	74.5 (66.6-82.6)	1.30 (0.76-2.21)	NS
	Black	53	91.3 (84.2-98.6)	**4.13 (1.25-13.68)**	**0.02**	72	86.7 (79.5-94.0)	**2.29 (1.11-4.73)**	**0.02**
**HSV-2**									
	White	592	14.8 (13.7-15.9)			823	16.0 (15.0-17.0)		
	Asian	16	13.1 (7.1-19.1)	1.14 (0.61-2.16)	NS	22	19.3 (12.1-26.5)	1.26 (0.65-2.44)	NS
	Black	28	48.3 (35.4-61.1)	**4.30 (2.12-8.75)**	**5.6x10^{-5}**	47	56.6 (45.9-67.3)	**5.77 (3.36-9.91)**	**2.1x10^{-10}**
**EBV**									
	White	3735	93.2 (92.4-93.9)			4903	95.6 (94.9-96.1)		
	Asian	118	96.7 (93.6-99.9)	4.03 (0.97-16.72)	NS	112	98.2 (95.8-100)	1.88 (0.45-7.86)	NS
	Black	58	100	NA	NA	81	97.6 (94.3-100)	2.59 (0.33-20.33)	NS
**CMV**									
	White	2195	54.8 (53.2-56.3)			2967	57.8 (56.5-59.2)		
	Asian	114	93.4 (89.1-97.8)	**10.81 (4.94-23.68)**	**2.67x10^{-9}**	103	90.4 (84.9-95.8)	**6.27 (3.08-12.76)**	**7.4x10^{-5}**
	Black	55	94.8 (89.1-100)	**9.76 (2.95-32.24)**	**1.9x10^{-4}**	77	92.8 (87.2-98.3)	**9.22 (3.89-21.82)**	**4.1x10^{-7}**
**KSHV**									
	White	329	8.2 (7.4-9.1)			372	7.3 (6.5-8.0)		
	Asian	11	9.0 (3.9-14.1)	1.23 (0.58-2.62)	NS	14	12.3 (6.3-18.3)	1.77 (0.86-3.63)	NS
	Black	19	32.8 (20.7-44.8)	**7.10 (3.45-14.54)**	**9.4x10^{-8}**	14	16.9 (8.8-24.9)	**3.00 (2.54-5.82)**	**1.2x10^{-3}**
**HBV**									
	White	90	2.2 (1.8-2.7)			62	1.2 (0.9-1.5)		
	Asian	19	15.6 (9.1-22.0)	**7.60 (3.70-15.64)**	**3.6x10^{-8}**	12	10.5 (4.9-16.2)	**9.24 (3.50-25.38)**	**9.1x10^{-6}**
	Black	21	36.2 (23.8-48.6)	**2.82 (12.72-62.63)**	**2.1x10^{-16}**	12	14.5 (6.9-22.0)	**8.59 (3.05-24.13)**	**4.6x10^{-5}**
**HPV-18**									
	White	72	1.8 (1.4-2.2)			159	3.1 (1.4-4.8)		
	Asian	5	4.1 (0.6-7.6)	2.90 (0.98-8.54)	NS	7	6.1 (4.4-7.8)	3.55 (1.47-8.57)	**4.8x10^{-3}**
	Black	2	3.4 (0-8.1)	3.96 (0.88-17.9)	NS	7	8.4 (6.7-10.1)	2.64 (1.07-6.52)	**0.03**
**JCV**									
	White	2422	60.4 (58.9-61.9)			2780	54.1 (52.8-55.5)		
	Asian	85	69.7 (61.5-77.8)	**1.68 (1.03-2.75)**	**0.03**	85	74.6 (66.6-82.6)	2.68 (1.52-4.71)	**6.3x10^{-4}**
	Black	41	70.7 (59.0-82.4)	**2.82 (1.15-6.88(**	**0.02**	55	66.3 (56.1-76.4)	1.57 (0.90-2.71)	NS
**BKV**									
	White	3849	96.0 (95.4-96.6)			4860	94.7 (94.1-95.3)		
	Asian	118	96.7 (93.6-99.9)	1.53 (0.37-6.37)	NS	107	93.9 (89.4-98.3)	0.70 (0.25-1.97)	NS
	Black	57	98.3 (94.9-100)	1.17 (0.16-8.81)	NS	83	100	NA	NA
**MCV**									
	White	2698	67.3 (65.8-68.8)			3360	65.5 (64.2-66.8)		
	Asian	88	72.1 (64.2-80.1)	1.28 (0.79-2.09)	NS	78	68.4 (59.9-77.0)	1.36 (0.80-2.30)	NS
	Black	49	84.5 (75.2-93.8)	2.12 (0.87-5.17)	NS	68	81.9 (73.6-90.2)	**2.47 (1.24-4.93)**	**0.01**
Tg									
	White	1121	28.0 (26.6-29.4)			1356	26.4 (25.2-27.6)		
	Asian	56	45.9 (37.1-54.7)	**2.78 (1.74-4.27)**	**1.17x10^{-5}**	44	38.6 (29.7-47.5)	**1.79 (1.09-2.94)**	**0.02**
	Black	31	53.4 (40.6-66.3)	**3.56 (1.76-7.20)**	**4.1x10^{-4}**	33	39.8 (29.2-50.3)	1.58 (0.92-2.71)	NS
***Hp*‡**									
	White	723	37.0 (34.8-39.1)			838	31.6 (29.9-33.4)		
	Asian	34	60.7 (47.9-73.5)	**2.81 (1.39-5.66)**	**3.9x10^{-3}**	33	57.9 (45.1-70.7)	**3.52 (1.72-7.24)**	**5.8x10^{-4}**
	Black	18	78.3 (61.4-95.1)	**22.27 (2.89-171.3)**	**2.9x10^{-3}**	33	73.3 (60.4-86.3)	**6.30 (2.98-13.36)**	**1.5x10^{-6}**
Ct									
	White	584	14.5 (13.5-15.6)			1265	24.6 (23.5-25.8)		
	Asian	34	27.9 (19.9-35.8)	**4.66 (2.73-7.94)**	**1.58x10^{-8}**	31	27.2 (19.0-35.4)	**1.84 (1.02-3.29)**	**0.04**
	Black	32	55.2 (42.4-68.0)	**5.63 (2.70-1.17)**	**4.21x10^{-6}**	56	67.5 (57.4-77.5)	**6.19 (3.41-11.23)**	**2.0x10^{-9}**

*: Odds ratio and p-values calculated using multivariable logistic regression reported after adjustment for age interval, TDI, LSP and sameSI. Significant associations after adjustment are highlighted in bold. NS: not significant.

†: White ethnic group used as reference in models (n_{males}=4009, $n_{females}$=5131) against Asian (n_{males}=122, $n_{females}$=114) and Black (n_{males}=58, $n_{females}$ =83).

‡: Only samples from week 1 were available giving at total number of individual in each category as White (n_{males}=1955, $n_{females}$=2649), Asian (n_{males}=56, $n_{females}$=57) and Black (n_{males}=23, $n_{females}$=45).

Supplementary Table 11: Unadjusted seroprevalence estimates and adjusted odds ratios of antibody prevalence against all tested infections in the UKB sample clustered by LSP and stratified by sex.

Agent	LSP[†]	Seropositive Males	Seroprevalence Males (95%CI)	Adjusted OR* Males (95%CI)	*P*-value* Males	Seropositive Females	Seroprevalence Females (95%CI)	Adjusted OR* Females (95%CI)	*P*-value* Females
**HSV-1**									
	0-1	528	60.1 (56.9-63.4)			995	64.2 (61.8-66.6)		
	2-4	674	66.8 (63.9-69.7)	**1.30 (1.07-1.59)**	**8.4 x10^{-3}**	1084	71.1 (68.8-73.4)	**1.39 (1.19-1.63)**	**3.5x10^{-5}**
	5-10	690	70.7 (67.8-73.6)	**1.60 (1.31-1.97)**	**6.2x10^{-6}**	804	74.3 (71.7-76.9)	**1.73 (1.44-2.07)**	**4.6x10^{-9}**
	>10	463	71.7 (68.2-75.1)	**1.71 (1.35-2.17)**	**8.0x10^{-6}**	260	72.0 (67.4-77.0)	**1.56 (1.19-2.03)**	**1.1x10^{-3}**
**HSV-2**									
	0-1	104	11.8 (9.7-14.0)			152	9.8 (8.3-11.3)		
	2-4	124	12.3 (10.3-14.3)	1.00 (0.75-1.34)	NS	226	14.8 (13.0-16.6)	**1.53 (1.22-1.91)**	**2.3x10^{-4}**
	5-10	135	13.8 (11.7-16.0)	1.16 (0.87-1.55)	NS	241	22.3 (19.8-24.8)	**2.55 (2.02-3.23)**	**2.9x10^{-15}**
	>10	137	21.2 (18.1-24.4)	**1.91 (1.42-2.59)**	**2.0x10^{-5}**	115	31.9 (27.0-36.7)	**4.30 (3.30-5.78)**	**3.8x10^{-22}**
**VZV**									
	0-1	825	94.0 (92.4-95.5)			1422	91.8 (90.4-93.2)		
	2-4	961	95.2 (93.9-96.6)	1.23 (0.81-1.89)	NS	1383	90.7 (89.2-92.1)	0.83 (0.65-1.08)	NS
	5-10	914	93.6 (92.1-95.2)	0.94 (0.62-1.42)	NS	989	91.4 (89.7-93.1)	0.91 (0.68-1.22)	NS
	>10	604	93.5 (91.6-95.4)	1.00 (0.63-1.58)	NS	325	90.0 (86.9-93.1)	0.78 (0.51-1.17)	NS
**EBV**									
	0-1	759	86.4 (84.2-88.7)			1423	91.9 (90.5-93.2)		
	2-4	934	92.6 (90.9-94.2)	**1.75 (1.27-2.42)**	**6.4x10^{-4}**	1476	96.8 (95.9-97.7)	**2.442 (1.71-3.43)**	**7.4x10^{-7}**
	5-10	943	96.6 (95.5-97.8)	**4.06 (2.68-6.14)**	**3.6x10^{-11}**	1069	98.7 (98.1-99.4)	**6.67 (3.52-11.53)**	**1.0x10^{-9}**
	>10	631	97.7 (96.5-98.8)	**5.80 (3.28-10.24)**	**1.4x10^{-9}**	358	99.2 (98.2-100)	**9.22 (2.86-29.71)**	**2.0x10^{-4}**
**CMV**									
	0-1	487	55.5 (52.2-59.8)			915	59.1 (56.6-61.5)		
	2-4	573	56.8 (53.7-59.8)	1.06 (0.87-1.28)	NS	915	60.0 (57.5-62.5)	1.12 (0.97-1.31)	NS
	5-10	556	57.0 (53.9-60.1)	1.12 (0.92-1.36)	NS	604	55.8 (52.9-58.8)	1.05 (0.88-1.24)	NS
	>10	328	50.8 (46.9-54.6)	0.92 (0.74-1.14)	NS	204	56.5 (51.4-61.6)	1.17 (0.92-1.51)	NS
**HHV-6**									
	0-1	792	90.2 (88.2-92.2)			1413	91.2 (89.8-92.6)		
	2-4	913	90.5 (88.7-92.3)	1.01 (0.73-1.39)	NS	1378	90.4 (88.9-91.8)	0.92 (0.71-1.18)	NS
	5-10	885	90.7 (88.9-92.5)	1.03 (0.75-1.43)	NS	972	89.8 (88.0-91.6)	0.83 (0.63-1.10)	NS
	>10	599	92.7 (90.7-94.7)	1.38 (0.93-2.06)	NS	323	89.5 (86.3-92.6)	0.82 (0.49-1.22)	NS
**HHV-7**									
	0-1	816	92.9 (91.2-94.6)			1478	95.4 (94.4-96.5)		
	2-4	913	90.5 (88.7-92.3)	**0.66 (0.47-0.94)**	**0.02**	1468	96.3 (95.3-97.2)	1.25 (0.87-1.80)	NS
	5-10	916	93.9 (92.3-95.4)	1.08 (0.73-1.59)	NS	1047	96.8 (95.7-97.8)	1.34 (0.87-2.08)	NS
	>10	611	94.6 (92.8-96.3)	1.13 (0.72-1.77)	NS	349	96.7 (94.8-98.5)	1.28 (0.67-2.46)	NS
**KSHV**									
	0-1	59	6.7 (5.1-8.4)			120	7.7 (6.4-9.1)		
	2-4	80	7.9 (6.3-9.6)	1.12 (0.78-1.60)	NS	126	8.3 (6.9-9.6)	1.07 (0.82-1.40)	NS
	5-10	91	9.3 (7.5-11.1)	1.29 (0.90-1.84)	NS	82	7.6 (6.0-9.2)	0.96 (0.71-1.31)	NS
	>10	57	8.8 (6.6-11.0)	1.15 (0.76-1.73)	NS	31	8.6 (5.7-11.5)	1.11 (0.72-1.72)	NS
**HBV**									
	0-1	19	2.2 (1.2-3.1)			16	1.0 (0.6-1.5)		
	2-4	27	2.7 (1.7-3.7)	1.06 (0.56-1.98)	NS	21	1.4 (0.8-2.0)	1.36 (0.70-2.68)	NS
	5-10	24	2.5 (1.5-3.4)	0.94 (0.49-1.82)	NS	18	1.7 (0.9-2.4)	1.77 (0.86-3.66)	NS
	>10	27	4.2 (2.6-5.7)	1.47 (0.74-2.89)	NS	7	1.9 (0.5-3.4)	2.02 (0.76-5.36)	NS
**HCV**									
	0-1	2	0.2 (0-0.5)			1	0.1 (0-0.2)		
	2-4	2	0.2 (0-0.5)	0.76 (0.10-5.58)	NS	3	0.2 (0-0.4)	NA	NA
	5-10	5	0.5 (0-1.0)	1.84 (0.34-10.01)	NS	1	0.2 (0-0.4)	NA	NA
	>10	4	0.6 (0-1.0)	1.86 (0.29-11.67)	NS	1	0.8 (0-1.8)	NA	NA
**HPV-16**									
	0-1	12	1.4 (0.6-2.1)			31	2.0 (1.3-2.7)		
	2-4	15	1.5 (0.7-2.2)	1.08 (0.49-2.37)	NS	73	4.8 (3.7-5.9)	**2.11 (1.67-3.26)**	**7.1x10^{-4}**
	5-10	26	2.7 (1.7-3.7)	1.89 (0.92-3.90)	NS	111	10.3 (8.5-12.1)	**4.53 (2.98-6.89)**	**1.7x10^{-12}**
	>10	34	5.3 (3.5-7.0)	**3.54 (1.72-7.31)**	**6.0x10^{-4}**	42	11.6 (8.3-14.9)	**5.11 (3.11-8.42)**	**1.4x10^{-10}**
**HPV-18**									
	0-1	16	1.8 (0.9-2.7)			25	1.6 (1.0-2.2)		
	2-4	12	1.2 (0.5-1.9)	0.60 (0.28-1.29)	NS	42	2.8 (1.9-3.6)	1.58 (0.95-2.63)	NS
	5-10	21	2.2 (1.2-3.1)	1.07 (0.54-2.12)	NS	55	5.1 (3.8-6.4)	**2.77 (1.67-4.58)**	**7.6x10^{-5}**
	>10	12	1.9 (0.8-2.9)	0.88 (0.39-1.99)	NS	29	8.0 (5.3-10.8)	**4.48 (2.51-8.02)**	**4.1x10^{-7}**
**HIV-1**									
	0-1	0	0			1	0.1 (0-0.2)		
	2-4	3	0.3 (0-0.6)	NA	NA	4	0.3 (0-0.5)	NA	NA
	5-10	0	0	NA	NA	0	0	NA	NA
	>10	3	0.5 (0-1.0)	NA	NA	0	0	NA	NA
**HTLV-1**									
	0-1	13	1.4 (0.6-2.3)			16	1.0 (0.5-1.5)		
	2-4	27	2.7 (1.7-3.7)	1.94 (0.99-3.82)	NS	19	1.0 (0.7-1.8)	1.34 (0.68-2.65)	NS
	5-10	25	2.6 (1.6-3.6)	1.97 (0.99-3.91)	NS	15	1.0 (0.7-2.1)	1.56 (0.74-3.32)	NS
	>10	9	1.4 (0.5-2.3)	1.19 (0.50-2.86)	NS	6	1.7 (0.3-3.0)	2.28 (0.85-6.11)	NS

**JCV**									
	0-1	535	60.9 (57.7-64.2)			848	54.7 (52.3-57.2)		
	2-4	602	59.7 (56.6-62.9)	0.93 (0.77-1.12)	NS	817	53.6 (51.1-56.1)	0.92 (0.80-1.07)	NS
	5-10	603	61.8 (58.7-64.8)	1.01 (0.83-1.23)	NS	600	55.4 (52.5-58.4)	0.96 (0.81-1.13)	NS
	>10	392	60.7 (56.9-64.4)	0.96 (0.77-1.20)	NS	200	55.4 (50.3-60.5)	0.95 (0.74-1.21)	NS
**BKV**									
	0-1	842	95.9 (94.6-97.2)			1472	95.0 (93.9-96.1)		
	2-4	971	96.2 (95.1-97.4)	1.06 (0.66-1.72)	NS	1448	95.0 (93.9-96.0)	0.91 (0.65-1.27)	NS
	5-10	942	96.5 (95.4-97.7)	1.16 (0.70-1.91)	NS	1036	95.7 (94.5-97.0)	0.97 (0.65-1.43)	NS
	>10	623	96.4 (95.0-97.9)	1.08 (0.61-1.91)	NS	348	96.4 (94.5-98.3)	1.14 (0.60-2.14)	NS
**MCV**									
	0-1	568	64.7 (61.5-67.9)			974	62.9 (60.5-65.3)		
	2-4	668	66.2 (63.3-69.1)	1.09 (0.90-1.33)	NS	987	64.7 (62.3-67.1)	1.04 (0.89-1.21)	NS
	5-10	682	69.9 (67.0-72.8)	**1.30 (1.06-1.59)**	**0.01**	754	70.0 (66.9-72.4)	**1.27 (1.07-1.51)**	**$7.3x10^{-3}$**
	>10	459	71.1 (67.6-74.5)	**1.40 (1.11-1.77)**	**$4.5x10^{-3}$**	257	71.2 (66.5-75.9)	**1.35 (1.04-1.75)**	**0.03**
Hp [‡]									
	0-1	142	33.5 (29.0-38.0)			262	33.7 (30.4-37.0)		
	2-4	195	40.0 (35.6-44.3)	1.29 (0.97-1.71)	NS	238	31.0 (27.7-34.3)	0.85 (0.68-1.07)	NS
	5-10	174	36.5 (32.2-41.0)	1.12 (0.84-1.50)	NS	183	31.1 (27.3-34.8)	0.88 (0.69-1.13)	NS
	>10	117	37.5 (32.1-42.9)	1.22 (0.88-1.69)	NS	48	25.4 (19.2-31.6)	0.67 (0.46-0.99)	NS
Tg									
	0-1	229	26.1 (23.2-29.0)			405	26.1 (24.0-28.3)		
	2-4	296	29.3 (26.5-32.1)	1.22 (0.98-1.50)	NS	404	26.5 (24.3-28.7)	1.04 (0.88-1.23)	NS
	5-10	288	29.5 (26.6-32.4)	1.24 (1.00-1.54)	0.05	310	28.7 (26.0-31.3)	1.20 (1.00-1.45)	0.05
	>10	176	27.2 (23.8-30.7)	1.15 (0.90-1.47)	NS	110	**30.5 (25.7-35.2)**	**1.34 (1.03-1.75)**	**0.03**
Ct									
	0-1	35	4.0 (2.7-5.3)			136	8.8 (7.4-10.2)		
	2-4	99	9.8 (8.0-11.6)	**2.54 (1.69-3.82)**	**$6.8x10^{-6}$**	301	19.7 (17.7-21.7)	**2.34 (1.88-2.92)**	**$5.7x10^{-14}$**
	5-10	166	17.0 (14.7-19.4)	**4.88 (3.30-7.21)**	**$2.2x10^{-15}$**	436	40.3 (37.4-43.2)	**6.51 (5.20-8.14)**	**$3.2x10^{-60}$**
	>10	216	33.4 (29.8-37.1)	**11.91 (8.00-17.71)**	**$2.2x10^{-34}$**	201	55.7 (50.6-60.8)	**12.57 (9.42-16.76)**	**$1.3x10^{-66}$**

[*]: Odds ratio (OR) and *P*-values for number of self-reported sexual partners in a multivariable logistic regression model after correcting for age, sex, TDI, self-reported ethnicity and sameSI. Significant associations are highlighted in bold. NA: did not converge owing to small numbers per category. NS: not significant.
[†]: 0-1 reported sexual partners was used as reference in all models. The total number of individuals per category were: males (878 in 0-1; 674 in 2-4; 976 in 5-10 and 646 in >10) and females (1549; 1525; 1082; 361).
[‡]: Only samples from week 1 were available giving at total number of individual in each category as 0-1 (n=424); 2-4 (488); 5-10 (477) and >10 (312) in males; and 0-1 (778); 2-4 (768); 5-10 (589) and >10 (189) in females.

Supplementary Table 12: Unadjusted seroprevalence estimates and adjusted odds ratios of antibody prevalence in the UKB sample clustered by sex and Townsend deprivation index quartile.

Infectious agents included represent those with significant (*P*<0.01) evidence of association based on crude estimates. The chi-squared test for trend was used to determine whether there was an overall upward or downward trend over quartiles within sex clusters.

Agent	TDI*	Sero-positive males	Seroprevalence males (95%CI)	Adjusted OR males (95%CI)	*P*-value† males	Trend *P*-value males	Sero-positive females	Seroprevalence females (95%CI)	Adjusted OR females (95%CI)	*P*-value† females	Trend *P*-value females
**HSV-1**											
	Q1	532	63.3 (60.0-66.5)				741	67.4 (64.7-70.2)			
	Q2	585	67.7 (64.6-70.8)	1.15 (0.92-1.43)	NS		727	67.6 (64.8-70.4)	1.03 (0.85-1.25)	NS	
	Q3	548	66.8 (63.6-70.1)	1.13 (0.91-1.41)	NS		763	68.4 (65.6-71.1)	1.10 (0.91-1.34)	NS	
	Q4	573	67.3 (64.2-70.5)	1.14 (0.91-1.42)	NS		794	73.1 (70.5-75.7)	**1.30 (1.06-1.58)**	**0.01**	
	Q5	693	77.7 (75.0-80.4)	**1.89 (1.49-2.41)**	**2.0x10^{-7}**	**7.9x10^{-9}**	809	77.6 (75.1-80.2)	**1.53 (1.23-1.89)**	**1.3x10^{-3}**	**4.3x10^{-9}**
**HSV-2**											
	Q1	107	12.7 (10.5-15.0)				153	13.9 (11.9-16.0)			
	Q2	99	11.4 (9.3-13.6)	0.90 (0.65-1.25)	NS		151	16.0 (13.9-18.2)	0.97 (0.74-1.26)	NS	
	Q3	123	15.0 (12.6-17.4)	1.19 (0.87-1.62)	NS		179	16.0 (13.9-18.2)	0.97 (0.93-1.55)	NS	
	Q4	134	15.7 (13.3-18.2)	1.23 (0.91-1.68)	NS		193	17.8 (15.5-20.0)	1.20 (0.91-1.52)	NS	
	Q5	190	21.3 (18.6-24.0)	1.32 (0.97-1.80)	NS	**2.1x10^{-8}**	237	22.7 (20.2-25.3)	1.21 (0.93-1.58)	NS	**4.4x10^{-9}**
**EBV**											
	Q1	776	92.3 (90.5-94.1)				1041	94.7 (93.4-96.0)			
	Q2	804	93.1 (91.4-94.8)	1.00 (0.67-1.49)	NS		1016	94.4 (93.1-95.8)	0.83 (0.55-1.25)	NS	
	Q3	761	92.8 (91.0-94.6)	1.00 (0.68-1.49)	NS		1066	95.5 (94.3-96.7)	1.18 (0.76-1.85)	NS	
	Q4	793	93.2 (91.5-94.9)	1.12 (0.74-1.71)	NS		1056	97.2 (96.3-98.2)	**1.73 (1.03-2.90)**	**0.04**	
	Q5	854	95.7 (94.4-97.1)	1.44 (0.91-2.29)	NS	**7.2x10^{-3}**	1007	96.6 (95.5-97.7)	1.02 (0.63-1.66)	NS	**6.6x10^{-4}**
**CMV**											
	Q1	454	54.0 (50.6-57.4)				635	57.8 (54.9-60.7)			
	Q2	480	55.6 (52.2-58.9)	1.07 (0.87-1.33)	NS		617	57.3 (54.4-60.3)	0.96 (0.79-1.15)	NS	
	Q3	469	57.2 (53.8-60.6)	1.17 (0.74-1.44)	NS		651	58.3 (55.4-61.2)	1.07 (0.89-1.30)	NS	
	Q4	476	55.9 (52.6-59.3)	1.06 (0.85-1.32)	NS		639	58.8 (55.9-61.8)	1.02 (0.85-1.24)	NS	
	Q5	546	61.2 (58.0-64.4)	1.22 (0.98-1.52)	NS	**4.9x10^{-3}**	673	54.6 (61.7-67.5)	1.12 (0.91-1.36)	NS	**1.7x10^{-3}**
**KSHV**											
	Q1	76	9.0 (7.1-11.0)				66	6.0 (4.6-7.4)			
	Q2	57	6.6 (4.9-8.3)	0.71 (0.47-1.06)	NS		71	6.6 (5.1-8.0)	0.99 (0.69-1.42)	NS	
	Q3	51	6.2 (4.6-7..9)	0.79 (0.53-1.18)	NS		85	7.6 (6.1-9.2)	1.19 (0.84-1.69)	NS	
	Q4	79	9.3 (7.3-11.2)	1.01 (0.69-1.47)	NS		103	9.5 (7.7-11.2)	**1.45 (1.03-2.04)**	**0.03**	
	Q5	109	12.2 (10.1-14.4)	1.17 (0.81-1.70)	NS	**2.2x10^{-3}**	85	8.2 (6.5-9.8)	1.29 (0.90-1.86)	NS	**4.5x10^{-3}**
**HBV**											
	Q1	14	1.7 (0.8-2.5)				17	1.5 (0.8-2.3)			
	Q2	25	2.9 (1.8-4.0)	1.73 (0.84-3.56)	NS		14	1.3 (0.6-2.0)	0.98 (0.44-2.21)	NS	
	Q3	17	2.0 (1.1-3.0)	1.18 (0.54-2.59)	NS		11	1.0 (0.4-1.6)	0.65 (0.26-1.60)	NS	
	Q4	27	3.2 (2.0-4.4)	1.12 (0.52-2.42)	NS		17	1.6 (0.8-2.3)	0.78 (0.34-1.82)	NS	
	Q5	58	6.5 (4.9-8.1)	1.57 (0.76-3.24)	NS	**1.6x10^{-7}**	38	3.6 (2.5-4.8)	1.31 (0.61-2.82)	NS	**6.4x10^{-4}**
Tg											
	Q1	227	27.0 (24.0-30.0)				274	24.9 (22.4-27.5)			
	Q2	230	26.6 (23.7-29.6)	0.91 (0.72-1.16)	NS		266	24.7 (22.1-27.3)	1.01 (0.82-1.25)	NS	
	Q3	221	27.0 (23.9-30.0)	0.99 (0.78-1.26)	NS		285	25.5 (23.0-28.1)	1.04 (0.84-1.28)	NS	
	Q4	259	30.4 (27.3-33.5)	1.12 (0.89-1.42)	NS		316	29.1 (26.4-31.8)	1.16 (0.94-1.43)	NS	
	Q5	301	33.7 (30.6-36.8)	1.23 (0.97-1.56)	NS	**3.4x10^{-4}**	331	31.8 (28.9-34.6)	**1.34 (1.08-1.66)**	**7.9x10^{-3}**	**3.0x10^{-5}**
Hp											
	Q1	134	30.9 (26.5-35.2)				152	25.7 (22.2-29.2)			
	Q2	153	35.2 (30.7-40.0)	1.10 (0.80-1.49)	NS		161	29.1 (25.3-32.9)	1.17 (0.87-1.56)	NS	
	Q3	144	38.4 (33.5-43.3)	1.21 (0.88-1.66)	NS		176	32.2 (28.3-36.0)	**1.48 (1.11-1.97)**	**7.2x10^{-3}**	
	Q4	155	38.6 (33.8-43.3)	1.14 (0.83-1.57)	NS		206	36.3 (32.4-40.3)	**1.84 (1.39-2.44)**	**2.3x10^{-5}**	
	Q5	206	48.6 (43.8-53.3)	**1.62 (1.18-2.22)**	**3.1x10^{-3}**	**1.6x10^{-7}**	230	42.8 (38.6-47.0)	**1.94 (1.45-2.60)**	**8.9x10^{-6}**	**4.3x10^{-11}**
Ct											
	Q1	105	12.4 (10.3-14.7)				217	19.7 (17.4-22.1)			
	Q2	104	12.0 (9.9-14.2)	0.86 (0.63-1.24)	NS		242	22.5 (20.0-25.0)	1.23 (0.96-1.57)	NS	
	Q3	132	16.1 (13.6-18.6)	1.32 (0.96-1.83)	NS		275	24.6 (22.1-27.2)	1.27 (1.00-1.61)	NS	
	Q4	150	17.6 (17.1-20.2)	1.17 (0.85-1.61)	NS		294	27.1 (24.4-29.7)	1.21 (0.95-1.54)	NS	
	Q5	186	20.9 (18.2-23.5)	1.09 (0.79-1.51)	NS	**1.1x10^{-8}**	363	34.8 (31.9-37.7)	**1.58 (1.24-2.01)**	**2.1x10^{-3}**	**2.5x10^{-16}**

*: TDI expressed in quintiles with quintile 1 (Q1) used as reference in models (males n=841; females 1099) compared to Q2 (864; 1076), Q3 (820; 1116), Q4 (851; 1086) and Q5 (892; 1042).

†: Odds ratio and *P*-value for age group in a multivariable logistic regression model after correcting for Townsend deprivation index, ethnic group and number of lifetime sexual partners. Significant associations are highlighted in bold. NS: not significant.

‡: Only samples from week 1 were available giving at total number of individual in each category as Q1 (males n = 434; females 592); Q2 (435; 553); Q3 (375; 549), Q4 (402; 567) and Q5 (424; 537).

Supplementary Table 13: Characteristics of human genetic variants available for analysis in the UKB subset.

Variant Tested	Chromosome	Base pair position*	Alternate allele	Ancestral allele	UKB INFO†	Model Used	Trait tested
rs9269910	6	32551350	A	T	NA	Additive	JCV serostatus
rs9269268	6	32454667	C	T	0.96	Additive	MCV serostatus
rs6927022	6	32612397	A	G	0.96	Additive	EBV EBNA-1

*: Base pair position in build 37 co-ordinates of the human genome.
†: INFO scores available for imputed variants. NA where variants were directly genotyped.

Supplementary Table 14: HLA alleles associated with tested quantitative antibody responses.

Variant Tested	UKB Frequency (%)	Beta	SE	P*	Trait tested
HLA-DRB1*15:01	0.14	-0.18	0.02	$4.0x10^{-28}$	Log10 JCV VP1 magnitude
HLA-DRB1*15:01	0.14	-0.20	0.02	$8.9x10^{-17}$	Log10 MCV VP1 magnitude
HLA-DQB1*02	0.15	-0.18	0.02	$1.5x10^{-13}$	Log10 EBV EBNA-1 magnitude
HLA-DRB1*04:04	0.05	0.08	0.02	1.1x10-5	Log10 EBV VCA magnitude

*: P-values calculated using linear regression adjusting for age and sex.

12. References

1. Waterboer T*, et al.* Multiplex human papillomavirus serology based on in situ-purified glutathione s-transferase fusion proteins. *Clinical chemistry* **51**, 1845-1853 (2005).

2. Waterboer T, Sehr P, Pawlita M. Suppression of non-specific binding in serological Luminex assays. *Journal of immunological methods* **309**, 200-204 (2006).

3. Sehr P, Muller M, Hopfl R, Widschwendter A, Pawlita M. HPV antibody detection by ELISA with capsid protein L1 fused to glutathione S-transferase. *J Virol Methods* **106**, 61-70 (2002).

4. Sehr P, Zumbach K, Pawlita M. A generic capture ELISA for recombinant proteins fused to glutathione S-transferase: validation for HPV serology. *Journal of immunological methods* **253**, 153-162 (2001).

5. Loimaranta V*, et al.* Comparison of multiplex-serology and ELISA based methods in detecting HPV16 L1 antibody responses in paired saliva and serum samples of healthy men. *J Virol Methods* **270**, 26-33 (2019).

6. Simon J*, et al.* Validation of an Epstein-Barr Virus Antibody Risk Stratification Signature for Nasopharyngeal Carcinoma by Use of Multiplex Serology. *Journal of clinical microbiology* **58**, (2020).

7. Brenner N*, et al.* Validation of Multiplex Serology detecting human herpesviruses 1-5. *PloS one* **13**, e0209379 (2018).

8. Brenner N*, et al.* Validation of Multiplex Serology for human hepatitis viruses B and C, human T-lymphotropic virus 1 and Toxoplasma gondii. *PloS one* **14**, e0210407 (2019).

9. Kranz LM, Gartner B, Michel A, Pawlita M, Waterboer T, Brenner N. Development and validation of HIV-1 Multiplex Serology. *Journal of immunological methods* **466**, 47-51 (2019).

10. Trabert B*, et al.* Antibodies Against Chlamydia trachomatis and Ovarian Cancer Risk in Two Independent Populations. *Journal of the National Cancer Institute*, pii: 5001107 (2018).

11. Bland JM, Altman DG. The use of transformation when comparing two means. *Bmj* **312**, 1153 (1996).

12. Koo TK, Li MY. A Guideline of Selecting and Reporting Intraclass Correlation Coefficients for Reliability Research. *J Chiropr Med* **15**, 155-163 (2016).

13. Michael KM*, et al.* Seroprevalence of 34 human papillomavirus types in the German general population. *PLoS pathogens* **4**, e1000091 (2008).

14. Combes JD*, et al.* Antibodies against high-risk human papillomavirus proteins as markers for invasive cervical cancer. *International journal of cancer Journal international du cancer* **135**, 2453-2461 (2014).

15. Heideman DA*, et al.* Human papillomavirus-16 is the predominant type etiologically involved in penile squamous cell carcinoma. *Journal of clinical oncology : official journal of the American Society of Clinical Oncology* **25**, 4550-4556 (2007).

16. Holzinger D*, et al.* Sensitivity and specificity of antibodies against HPV16 E6 and other early proteins for the detection of HPV16-driven oropharyngeal squamous cell carcinoma. *International journal of cancer Journal international du cancer* **140**, 2748-2757 (2017).

17. Kreimer AR*, et al.* Human papillomavirus antibodies and future risk of anogenital cancer: a nested case-control study in the European prospective investigation into cancer and nutrition study. *Journal of clinical oncology : official journal of the American Society of Clinical Oncology* **33**, 877-884 (2015).

18. Kreimer AR*, et al.* Evaluation of human papillomavirus antibodies and risk of subsequent head and neck cancer. *Journal of clinical oncology : official journal of the American Society of Clinical Oncology* **31**, 2708-2715 (2013).

19. Kreimer AR*, et al.* Kinetics of the Human Papillomavirus Type 16 E6 Antibody Response Prior to Oropharyngeal Cancer. *Journal of the National Cancer Institute* **109**, (2017).

20. Gossai A*, et al.* Prospective Study of Human Polyomaviruses and Risk of Cutaneous Squamous Cell Carcinoma in the United States. *Cancer epidemiology, biomarkers & prevention : a publication of the American Association for Cancer Research, cosponsored by the American Society of Preventive Oncology* **25**, 736-744 (2016).

21. Kjaerheim K*, et al.* Absence of SV40 antibodies or DNA fragments in prediagnostic mesothelioma serum samples. *International journal of cancer Journal international du cancer* **120**, 2459-2465 (2007).

22. Robles C*, et al.* Seroreactivity against Merkel cell polyomavirus and other polyomaviruses in chronic lymphocytic leukaemia, the MCC-Spain study. *J Gen Virol* **96**, 2286-2292 (2015).

23. Bycroft C*, et al.* The UK Biobank resource with deep phenotyping and genomic data. *Nature* **562**, 203-209 (2018).

24. Hammer C*, et al.* Amino Acid Variation in HLA Class II Proteins Is a Major Determinant of Humoral Response to Common Viruses. *Am J Hum Genet* **97**, 738-743 (2015).

25. Yang J, Lee SH, Goddard ME, Visscher PM. GCTA: a tool for genome-wide complex trait analysis. *Am J Hum Genet* **88**, 76-82 (2011).

26. Loh PR*, et al.* Efficient Bayesian mixed-model analysis increases association power in large cohorts. *Nat Genet* **47**, 284-290 (2015).

27. the International Multiple Sclerosis Genetics C. Class II HLA interactions modulate genetic risk for multiple sclerosis. *Nat Genet* **47**, 1107-1113 (2015).

28. Jia X*, et al.* Imputing amino acid polymorphisms in human leukocyte antigens. *PloS one* **8**, e64683 (2013).

29. Purcell S*, et al.* PLINK: a tool set for whole-genome association and population-based linkage analyses. *Am J Hum Genet* **81**, 559-575 (2007).

30. Han B, Eskin E. Random-effects model aimed at discovering associations in meta-analysis of genome-wide association studies. *Am J Hum Genet* **88**, 586-598 (2011).

31. Lee SH, Yang J, Goddard ME, Visscher PM, Wray NR. Estimation of pleiotropy between complex diseases using single-nucleotide polymorphism-derived genomic relationships and restricted maximum likelihood. *Bioinformatics* **28**, 2540-2542 (2012).

32. Bassig BA*, et al.* Serologic markers of viral infection and risk of non-Hodgkin lymphoma: A pooled study of three prospective cohorts in China and Singapore. *International journal of cancer Journal international du cancer* **143**, 570-579 (2018).

33. Engdahl E*, et al.* Increased Serological Response Against Human Herpesvirus 6A Is Associated With Risk for Multiple Sclerosis. *Front Immunol* **10**, 2715 (2019).

34. Freuer D*, et al.* Seropositivity of selected chronic infections and different measures of obesity. *PloS one* **15**, e0231974 (2020).

35. Dondog B*, et al.* Hepatitis C Virus Seroprevalence in Mongolian Women Assessed by a Novel Multiplex Antibody Detection Assay. *Cancer epidemiology, biomarkers & prevention : a publication of the American Association for Cancer Research, cosponsored by the American Society of Preventive Oncology* **24**, 1360-1365 (2015).

36. Petter A*, et al.* Specific serum IgG, IgM and IgA antibodies to human papillomavirus types 6, 11, 16, 18 and 31 virus-like particles in human immunodeficiency virus-seropositive women. *J Gen Virol* **81**, 701-708 (2000).

37. Hulstein SH*, et al.* Differences in Chlamydia trachomatis seroprevalence between ethnic groups cannot be fully explained by socioeconomic status, sexual healthcare seeking behavior or sexual risk behavior: a cross-sectional analysis in the HEalthy LIfe in an Urban Setting (HELIUS) study. *BMC infectious diseases* **18**, 612 (2018).

38. Wills GS*, et al.* Pgp3 antibody enzyme-linked immunosorbent assay, a sensitive and specific assay for seroepidemiological analysis of Chlamydia trachomatis infection. *Clinical and vaccine immunology : CVI* **16**, 835-843 (2009).

39. Michel A, Waterboer T, Kist M, Pawlita M. Helicobacter pylori multiplex serology. *Helicobacter* **14**, 525-535 (2009).
