# Supplementary material for: Identification of host–pathogen-disease relationships using a scalable multiplex serology platform in UK Biobank
Source: Nat Commun. 2022 Apr 5;13:1818. doi: 10.1038/s41467-022-29307-3 (PMC8983701; doi:10.1038/s41467-022-29307-3)
Supplement: Supplementary file 3 — Reporting Summary [file 41467_2022_29307_MOESM3_ESM.pdf]

nature research

Corresponding author(s): Alexander J Mentzer

Last updated by author(s): 2022/02/16

Reporting Summary

Nature Research wishes to improve the reproducibility of the work that we publish. This form provides structure for consistency and transparency in reporting. For further information on Nature Research policies, see our Editorial Policies and the Editorial Policy Checklist.

Statistics

For all statistical analyses, confirm that the following items are present in the figure legend, table legend, main text, or Methods section.

n/a	Confirmed	
☐	☒	The exact sample size (*n*) for each experimental group/condition, given as a discrete number and unit of measurement
☐	☒	A statement on whether measurements were taken from distinct samples or whether the same sample was measured repeatedly
☐	☒	The statistical test(s) used AND whether they are one- or two-sided<br>*Only common tests should be described solely by name; describe more complex techniques in the Methods section.*
☐	☒	A description of all covariates tested
☐	☒	A description of any assumptions or corrections, such as tests of normality and adjustment for multiple comparisons
☐	☒	A full description of the statistical parameters including central tendency (e.g. means) or other basic estimates (e.g. regression coefficient) AND variation (e.g. standard deviation) or associated estimates of uncertainty (e.g. confidence intervals)
☐	☒	For null hypothesis testing, the test statistic (e.g. *F*, *t*, *r*) with confidence intervals, effect sizes, degrees of freedom and *P* value noted<br>*Give P values as exact values whenever suitable.*
☒	☐	For Bayesian analysis, information on the choice of priors and Markov chain Monte Carlo settings
☒	☐	For hierarchical and complex designs, identification of the appropriate level for tests and full reporting of outcomes
☐	☒	Estimates of effect sizes (e.g. Cohen's *d*, Pearson's *r*), indicating how they were calculated

Our web collection on statistics for biologists contains articles on many of the points above.

Software and code

Policy information about availability of computer code

Data collection	All data used for analysis in this publication were derived from the data available in UK Biobank, described and explorable within their online Data Showcase (http://www.ukbiobank.ac.uk/data-showcase/).
Data analysis	Data was analysed within SAS 9.4, R (versions 3.5.0, 3.5.1 and 3.3.1), or within a suite of genetic analysis packages including PLINK 1.9, GCTA 1.26.0, SNP2HLA v1.0.2, BOLT-LMM v2.3.1 or METASOFT v2.0.1.

For manuscripts utilizing custom algorithms or software that are central to the research but not yet described in published literature, software must be made available to editors and reviewers. We strongly encourage code deposition in a community repository (e.g. GitHub). See the Nature Research guidelines for submitting code & software for further information.

Data

Policy information about availability of data

All manuscripts must include a data availability statement. This statement should provide the following information, where applicable:

- Accession codes, unique identifiers, or web links for publicly available datasets
- A list of figures that have associated raw data
- A description of any restrictions on data availability

All data are available through the UKB Access Management System (https://bbams.ndph.ox.ac.uk/ams/) as described in the following link: https://biobank.ndph.ox.ac.uk/showcase/label.cgi?id=51428
A full list of data fields and identifiers are available in the Supplementary Materials. The antibody data are available in Category 1307.
The International Multiple Sclerosis dataset was available through the authors of the following paper: the International Multiple Sclerosis Genetics C. Class II HLA interactions modulate genetic risk for multiple sclerosis. Nat Genet 47, 1107-1113 (2015).

Field-specific reporting

Please select the one below that is the best fit for your research. If you are not sure, read the appropriate sections before making your selection.

- [x] Life sciences
- [ ] Behavioural & social sciences
- [ ] Ecological, evolutionary & environmental sciences

For a reference copy of the document with all sections, see nature.com/documents/nr-reporting-summary-flat.pdf

Life sciences study design

All studies must disclose on these points even when the disclosure is negative.

Sample size	A sample size of 10,000 was chosen so that we would expect to observe at least 10 episodes of seroprevalence for rare infections (up to 0.1% prevalence in the UK) without making an assumption about the true underlying prevalence using existing literature.
Data exclusions	Of the total 10,110 serum samples assayed, 29 samples (0.3%) were excluded from analyses (1 highly viscous serum, 8 pipetting errors, 8 were incorrectly diluted and 11 with insufficient bead counts at the reading step) or samples initially destined for repeat assessment samples invalidated due to other errors (n=2). All remaining samples were included in downstream analyses.
Replication	Of the remaining samples 9,695 represented study participants, 107 were blind-spiked duplicates, and 277 were repeat assessment encounter samples (n=277). The complete analyses of these duplicates are included in the manuscript and Supplementary Materials. We observed excellent levels of coefficients of variation in he blind-spiked duplicates when looking at seropositives only (<=12%).
Randomization	The 10,000 serum samples were selected from the total UKB sample-set at random using the sample extraction methods described in the document that may be found here: https://biobank.ndph.ox.ac.uk/ukb/refer.cgi?id=5636.
Blinding	Formal blinding was not required in our proposed study design. Measuring the serological antibody responses would generate novel data and the samples were always completely anonymised therefore blinding was inherent in the study design.

Reporting for specific materials, systems and methods

We require information from authors about some types of materials, experimental systems and methods used in many studies. Here, indicate whether each material, system or method listed is relevant to your study. If you are not sure if a list item applies to your research, read the appropriate section before selecting a response.

Materials & experimental systems

n/a	Involved in the study	
[ ]	[x]	Antibodies
[x]	[ ]	Eukaryotic cell lines
[x]	[ ]	Palaeontology and archaeology
[x]	[ ]	Animals and other organisms
[ ]	[x]	Human research participants
[x]	[ ]	Clinical data
[x]	[ ]	Dual use research of concern

Methods

n/a	Involved in the study	
[x]	[ ]	ChIP-seq
[x]	[ ]	Flow cytometry
[x]	[ ]	MRI-based neuroimaging

Antibodies

Antibodies used	The only commercially available antibody used in the study was the secondary antibody, a biotinylated goat anti-human IgG/IgM/IgA antibody (109-065-064, Jackson ImmunoResearch).
Validation	No primary antibody directed against viral or bacterial pathogens was used in the study.

April 2020

Human research participants

Policy information about studies involving human research participants

Population characteristics	The baseline characteristics of the 9,695 individuals included in the study are provided in Table 1 of the manuscript.
Recruitment	The recruitment of UK Biobank has been extensively described and discussed in the general literature. Briefly, half a million men and women aged 40-69 years attended one of 22 UKB assessment centres located throughout England, Scotland and Wales between 2006 and 2010. All participants completed a touchscreen questionnaire, verbal interview and had a range of physical measurements and blood, urine and saliva samples taken for long-term storage. A subset of 20,000 individuals attended a repeat assessment between 2012 and 2013. For this study, serum samples from 9,695 UKB participants were selected at random and assayed using the final Multiplex Serology panel.
Ethics oversight	The ethical approval for UK Biobank is extensively detailed and described online: https://www.ukbiobank.ac.uk/the-ethics-and-governance-council/<br>UK Biobank has approval from the North West Multi-centre Research Ethics Committee (UK) and informed consent was obtained from all participants.<br>This work was undertaken under project code 43920.

Note that full information on the approval of the study protocol must also be provided in the manuscript.
